# Stunning methods in aquaculture slaughter and their implications for fish welfare

**DOI:** 10.7717/peerj.21258

**Published:** 2026-05-18

**Authors:** Helen Lambert, Albin Gräns, Lynne U. Sneddon, Keri Tietge, Michelle Sinclair

**Affiliations:** 1 Animal Welfare Consultancy, Kingsteignton, Devon, United Kingdom; 2 Department of Applied Animal Science and Welfare, Swedish University of Agricultural Sciences, Gothenberg, Sweden; 3 Department of Biology and Environmental Sciences, University of Gothenburg, Gothenburg, Sweden; 4 Eurogroup for Animals, Brussels, Belgium; 5 A World of Good Initiative Inc, Santa Fe, Mexico, United States of America; 6 School of Veterinary Sciences, University of Queensland, Brisbane, Australia

**Keywords:** Humane slaughter, Consciousness indicators, Electroencephalography (EEG), Animal-based measures (ABMs), Species-specific welfare, Mis-stun rate, Physiological stress response, Legislative frameworks, Operator training, Technological innovation

## Abstract

The majority of fish farmed in aquaculture systems around the world are not stunned during slaughter, exposing them to considerable pain and distress. When stunning methods are applied, there is a lack of a comprehensive understanding of the welfare outcomes, including each stunning method’s reliability and accuracy. This review critically evaluates the stunning methods predominantly used in aquaculture systems in Europe, and more widely. Studies were included if they contained discernible information on fish welfare indicators, and these were synthesised and assessed against welfare factors spanning the pre-stunning, induction, and loss-of-consciousness phases. The results reveal significant variability across methods, with no single current method presenting itself without significant welfare concerns. Gas methods are recognised as highly aversive and painful for fish and may induce unconsciousness that is delayed in onset and not always sustained. In contrast, electrical and percussive methods can provide rapid unconsciousness when delivered with sufficient energy and precision, but their effectiveness often remains highly dependent on species, size, and operational parameters. A key limitation across all methods is the lack of validated species-specific indicators of consciousness that can be relied upon in high-throughput commercial settings. This gap is compounded by a shortage of research assessing the effectiveness of stunning methods under real-world, commercial conditions. To support progress and seek improved fish welfare outcomes for the millions slaughtered each year in the European Union, and the billions slaughtered globally, we propose welfare-based criteria as an evaluative framework to guide the refinement of existing methods and the development of novel approaches. These conceptual criteria highlight principal areas for improvement, further research, and technological development. Currently, there are many gaps in knowledge regarding the effectiveness of stunning methods, the specific practices used, and the welfare impact they have on fish. Addressing these gaps requires further scientific research to provide a stronger evidence base across species and production systems. Improving the welfare of farmed fish during slaughter will therefore depend on combining rigorous research with technological innovation, robust commercial validation, and effective consciousness monitoring and reporting in real-world contexts, alongside clear legislation, enforceable guidelines, and regular inspection to ensure stunning methods are correctly applied, and welfare is safeguarded.

## Introduction

The aquaculture industry is a rapidly expanding sector. In terms of biomass produced, it has now surpassed catch fisheries as the main producer of aquatic animals (FAO, 2024). Despite this enormous growth, efforts to improve the welfare status of farmed fish have lagged (Browning, 2023; Pietsch, 2025). Given the very large numbers of animals killed in aquaculture, from an ethical perspective, scientific, commercial, and policy attention must be directed to understanding how to manage farmed fish humanely up to the point of death. Guidelines for terrestrial animals demand that animals are stunned and rendered unconscious before the actual killing method, and that the animal should remain unconscious until death occurs (EFSA, 2004). Therefore, while stunning before killing is a standard component of slaughter for terrestrial farmed animals in Europe and other highly developed nations, it is not universally applied to fish. The vast majority of fish produced in aquaculture globally are killed without stunning, using methods such as ice slurry and asphyxiation, which cause a prolonged and painful death (Clemente et al., 2023; De la Rosa, Castro & Ginés, 2021; Mercogliano et al., 2024).

Where stunning methods are applied, their reliability and accuracy can vary considerably, and the lack of validated species-specific indicators of unconsciousness further hinders the issue (Daskalova et al., 2016; Gräns et al., 2016; Retter et al., 2018). These challenges are compounded by species-specific differences in physiology and stress tolerance. For example, African sharptooth catfish (*Clarias gariepinus*) and European eels (*Anguilla anguilla*) display distinct physiological and behavioural responses compared with salmonids, which complicates the generalisation of welfare outcomes across species (Brijs et al., 2021; Verheijen & Flight, 1997). Considering the primary requirement of effective stunning is that animals are rendered unconscious immediately and remain so until death (EFSA, 2004; Humane Slaughter Association (HSA), 2016; World Organisation for Animal Health (WOAH), 2015), failure to achieve these requirements risks exposing fish to pain and distress during the subsequent handling and killing procedures.

As awareness of these concerns has grown, surveys indicate that many consumers across different regions express a preference for animals to be stunned unconscious before slaughter, even in nations that scarcely practice stunning (Sapience, 2024; Sinclair et al., 2023). Furthermore, the issue has gained political traction in the European Union (EU), and in some countries, such as Norway, stunning prior to killing is already a mandatory part of the slaughter process (Animal Welfare Act, LOV-2009-06-19-97, §12, 2009). In 2020, the European Commission announced that it would revise EU animal welfare legislation to align with the latest scientific evidence, specifically referencing slaughter as a priority area (European Commission, 2020a; European Commission, 2020b). More recently, in its 2023 Inception Impact Assessment, the Commission proposed introducing species-specific slaughter provisions for the five most farmed fish species: Atlantic salmon (*Salmo salar*), common carp (*Cyprinus carpio*), rainbow trout (*Oncorhynchus mykiss*), European sea bass (*Dicentrarchus labrax*), and gilthead sea bream (*Sparus aurata*) (European Commission, 2023). However, at present, neither the timeline nor the scope of these provisions has been defined, leaving most fish across the EU largely unprotected at the point of slaughter.

With this in mind, understanding the full implications of the different stunning methods used in aquaculture and identifying areas requiring further research is critical to ensure meaningful welfare progress. Understanding these implications also provides an opportunity for innovative commercial enterprises to evaluate their protocols and policies with the aim of futureproofing their operations against forthcoming legislation. This review, therefore, aims to critically evaluate the stunning methods currently employed in aquaculture against key welfare concerns relating to their reliability, efficacy, and capacity to minimise pain, distress, and suffering. In doing so, it seeks to highlight the strengths and weaknesses of each method, identify areas in need of further development, and consequently, assess the welfare implications for the estimated 640 million fish slaughtered in the EU each year, and the billions of fish globally (Carpenter, Vanderzwalmen & Lambert, 2025). To achieve this, the review adopts a two-stage process, first identifying the key welfare concerns relevant to stunning, and then evaluating each stunning method against these concerns.

## Methods—Identification of Key Factors Influencing Welfare at Stunning

Using the relevant stages of stunning as a framework (*i.e.,* pre-stunning, stunning induction, loss of consciousness, and risk of recovery before death), we performed a literature review to identify the key welfare concerns for each stage. This exploratory approach allowed us to utilise both relevant studies and industry literature to determine the key welfare issues arising at each point of the process. To this end, searches were performed using both Google Scholar and Google, focusing on literature published between 1990 and 2025 relevant to commercial aquaculture systems. Literature on welfare impacts was reviewed from 1990 onwards, as earlier work remains essential for understanding the physiological, behavioural, and affective responses underpinning welfare during stunning, which are not dependent on recent technological developments. We used the following combinations of keywords for the search: “Aquaculture” AND “Stunning” AND “Welfare” AND “Stunning induction” OR “Pre-stunning” OR “Unconsciousness” OR “Reversibility” OR “Stunning indicators” OR “Consciousness indicators”, to guide the literature search. Literature at this stage was included if it contained discernible information relating to fish welfare outcomes, irrespective of methodological rigour, as the purpose was to map the key concerns rather than to evaluate study quality. Therefore, where peer-reviewed literature was limited, grey literature was included to supplement data on commercial use or observed welfare outcomes.

## Key Factors Influencing Welfare at Stunning

There are numerous factors to consider when evaluating the effectiveness and welfare impact of stunning methods used in aquaculture. This section considers the key welfare considerations derived from the literature search that are used to assess each stunning method. In total, 37 articles were found that included relevant information regarding fish welfare outcomes during the pre-stunning phase; 23 for the induction phase, and 18 applicable to loss of consciousness and risk of recovery. In total, 71 individual papers and reports were used to determine the following key factors affecting welfare at stunning.

### Pre-stunning phase

In the minutes or hours before stunning, fish are often subjected to multiple stressors, including crowding, handling, and air exposure. These stressors can have a cumulative, compounding effect on welfare, diminishing the fish’s ability to cope with additional stressors and sometimes culminating in death (Brijs et al., 2018; Lange et al., 2018; Petitjean et al., 2019). While such stressors are often accepted as part of the stunning process (De la Llave-Propín et al., 2025; Welfarm, 2023), their prevalence highlights the need for slaughter systems that actively minimise or eliminate them to safeguard fish welfare (Barreto et al., 2022; Hjelmstedt et al., 2021). Importantly, both the stress status of the fish and the environmental conditions during this pre-stunning phase may also influence the effectiveness of the subsequent stunning method, affecting how reliably unconsciousness is induced and maintained (Bowman et al., 2020). Thus, the pre-stunning phase should be considered not only a welfare concern in itself, but also a key determinant of the overall outcome of a stunning procedure, particularly when the stunning methods dictate or negate practices such as crowding or manual handling.

### Crowding

Crowding is a typical component of slaughter practices in commercial aquaculture systems, as fish must be gathered into high densities to allow capture and transfer for stunning (Stien et al., 2024; Villarroel & Lambooij, 2022). Whilst the duration of crowding before stunning is dependent on multiple factors, including the farm’s size and layout, the welfare risks are consistent (Stien et al., 2024). For instance, studies show that even a brief period of crowding can cause physical damage, including scale, skin, and eye damage, fractures and organ rupture, physiological and behavioural signs of stress, hypoxia, negative states such as fear and distress, and mortality (EFSA, 2009a; EFSA, 2009b; Erikson et al., 2016; Lines & Spence, 2012; Noble et al., 2018; Stien et al., 2024). Even when fish appear to swim normally, they may still experience rising cortisol levels indicative of stress, so behaviour is not always the most reliable indicator for measuring the impact of crowding on fish welfare (Erikson et al., 2016). This discrepancy arises because physiological stress responses can be activated and sustained even when fish suppress overt behavioural signs, meaning that apparently normal swimming does not necessarily equate to good welfare.

Some technical innovations, such as low-stress transfer systems, aim to reduce crowding impacts by using pumping mechanisms to transport fish continuously, but their effectiveness across species and farm designs remains variable, leaving crowding a key welfare concern (Føre et al., 2018; Stien et al., 2024; Welfarm, 2023). Consequently, crowding is widely considered to be a significant cause of welfare issues (Daskalova, 2019; De la Llave-Propín et al., 2025; Hjelmstedt et al., 2021) and is therefore included as a key welfare factor in this review.

#### Handling

Handling, whether by human operators or mechanical systems, is a routine part of slaughter practices in aquaculture, and a recognised welfare concern (De la Rosa, Castro & Ginés, 2021; European Commission, 2020c; Størkersen et al., 2021). Manual handling methods, including brailing, netting, and direct contact with humans, can cause damage to the fish’s protective mucous layer, fins, and scales, elicit fear responses, cause compression, and expose fish to air (Braithwaite & Boulcott, 2007; Breen et al., 2020; Cook et al., 2015; Noble et al., 2012; Powell, 2021). Mechanical handling systems, including pumps and conveyors, can also cause injuries from abrasive surfaces, crowding, and abrupt pressure changes (Erikson et al., 2016; Xu et al., 2020). Pumps are considered inherently stressful for fish, yet are viewed as preferable alternatives to other methods in the absence of a truly humane method (Welfarm, 2023).

Whilst the negative impacts of handling on physical and mental well-being are well-documented (Barreto et al., 2022; Brown & Dorey, 2019; Mercogliano et al., 2024; Powell, 2021), they are often overlooked or combined with other pre-slaughter stressors in the assessment of stunning methods (Brijs et al., 2018; Matos et al., 2010). Furthermore, handling practices remain highly variable and largely unregulated, negatively impacting welfare outcomes (Giménez-Candela, Saraiva & Bauer, 2020; Toni et al., 2019), making handling a critical welfare factor in this review.

#### Air exposure

Some stunning methods require the fish to be removed from the water whilst still conscious, the duration of which depends on the design, type, and layout of the equipment, the size and location of the farm, the operators, and the species of fish involved. For most fish species, even a brief period of air exposure can cause significant physiological stress (Schuck-Paim et al., 2025). For example, in rainbow trout, brief air exposure following exhaustive exercise exacerbates physiological disturbances, including greater extracellular acidosis, elevated blood lactate, and impaired blood gas status (Ferguson & Tufts, 1992). These effects translate into reduced survival: after 12 h, only 62% of trout survived a 30 s air exposure, and 28% survived a 60 s exposure, compared with 88% survival in exercised-only fish and 10% in unexercised controls.

Most species show aversion to air exposure, displaying escape attempts and vigorous movements (Robb & Kestin, 2002; Schuck-Paim et al., 2025), and will avoid locations where they have previously experienced it (Millot et al., 2014). In gilthead seabream, even brief exposure induces neuromolecular states thought to be indicative of negative emotions (Cerqueira et al., 2017). An analysis performed using the Welfare Footprint Framework—a method that quantifies the magnitude, duration, and intensity of negative welfare states—drawing on seven lines of evidence regarding the experience of fish during initial air exposure, concluded that fish likely experience ‘disabling’ or ‘excruciating’ levels of negative affective experience when exposed to air, even for a brief period (Schuck-Paim et al., 2025). Air exposure is therefore considered an essential welfare factor for assessing the relative performance of different stunning methods.

### Stunning induction

Stunning induction is the phase where fish are first exposed to the stunning method, before losing consciousness. Welfare concerns arise if the method is aversive or causes painful physical trauma before the fish is rendered unconscious. This section considers how fish may exhibit behavioural signs of aversion during the induction of stunning, the physiological stress response, and the potential for physical harm. To separate the welfare impacts, mis-stuns, where fish are not effectively stunned in the first instance, are covered in ‘Accuracy and reliability, and the risk of mis-stuns’.

#### Behavioural signs of aversion

Behavioural signs of aversion are a key indication that the fish is experiencing unnecessary pain and distress as a result of the stunning method, or that they have been ineffectively stunned. Behavioural signs of aversion to pain and other negative experiences are increasingly documented across a wide range of fish species, providing robust evidence of sentience and their capacity to suffer (Braithwaite & Boulcott, 2007; Readman et al., 2013; Sneddon, 2009; Sneddon et al., 2014). Therefore, these behavioural indicators provide a valuable basis for interpreting the welfare impacts of stunning procedures. For example, escape behaviour is associated with the experience of pain, fear, and active avoidance in fish (Martins et al., 2011; Yue, Moccia & Duncan, 2004), and is widely reported across multiple farmed species (*e.g.*, salmon, sea bass, trout, tilapia, and carp (Erikson, 2011; Pedrazzani et al., 2020; Rahmanifarah, Shabanpour & Sattari, 2011; Rucinque et al., 2023; Zampacavallo et al., 2015).

Not all species will respond to noxious stimuli the same way, and some, such as benthic or less mobile species, may not show overt signs of distress (Braithwaite & Boulcott, 2007; Domenici & Hale, 2019; Dunlop, Millsopp & Laming, 2006; Reilly et al., 2008; Sneddon & Roques, 2023). For instance, escape behaviour can be restricted or slower in benthic or less mobile species, even when suffering is likely (Domenici & Hale, 2019). Similarly, electro-immobilisation may suppress movement without inducing unconsciousness (EFSA, 2004; Gräns et al., 2016; Kestin, Wotton & Adams, 1995), thus a lack of behavioural response does not necessarily indicate an absence of suffering (see ‘Conflicting findings between indicators of stunning success’).

Nevertheless, whilst the absence of aversive behaviour does not necessarily mean a method is not distressing, behaviours such as struggling and escape attempts are clear indicators of compromised welfare. For this reason, behavioural signs of aversion are included as a welfare factor in this review framework.

#### Physiological stress response

Due to the nature of most slaughter methods, fish are exposed to a range of stressors prior to stunning, such as crowding and handling. These can elicit a physiological stress response in the fish, but this response may be more pronounced in methods that induce a more deleterious impact on welfare.

Cortisol is the primary glucocorticoid (a hormone associated with stress) in fish. It is widely reported in studies, as it provides a systematic, quantifiable index that can be used to compare different processes (Gräns et al., 2016; Jung-Schroers et al., 2020; Sadoul & Geffroy, 2019). However, cortisol levels are also influenced by multiple factors, including handling, environment, and the timing of sampling, therefore the results can be difficult to interpret in the context of stunning (Gräns et al., 2016). When used alongside other welfare indicators, physiological stress responses such as cortisol levels provide valuable insights into the relative welfare impacts of different stunning methods and are therefore included as a welfare factor in this review.

#### Physical trauma

Fish may undergo physical trauma in the pre-stunning phase as a result of handling and crowding processes. Fish may also incur physical trauma from the stunning procedures, whether this is as a direct result of the method, such as in percussive stunning, or due to a fault with the stunning method. For instance, fish may suffer damage, including fractures, haemorrhages, bruising, scale loss, and other injuries, either as a result of behavioural responses to the stunning process, direct contact with the equipment, or exposure to electrical current (Anders et al., 2019; González-Garoz et al., 2025; Nordgreen et al., 2008; Retter et al., 2018).

The welfare impact of physical trauma caused by the stunning method depends on whether the stun induces immediate unconsciousness that lasts until the fish is dead. If the stun fails to do so, whether because of a mis-stun, the method being non-immediate, or the fish regaining consciousness, then the fish may experience considerable pain, distress, and suffering (Lines & Spence, 2012). Therefore, the likelihood of a method causing physical trauma is important to consider, as is the probability that the fish will experience any welfare impact as a result.

### Loss of consciousness and recovery risk

A critical consideration for stunning methods is their efficacy and efficiency at inducing unconsciousness and maintaining it until death. This section outlines the key factors relevant to evaluating this process, including the onset and duration of unconsciousness, the welfare implications of an ineffective or reversible stun, and the use of indicators to establish unconsciousness.

#### Time to unconsciousness

Insensibility is considered in the Council Regulation (EC) No 1099/2009 as an absence of reflexes or reactions to stimuli such as sound, odour, light or physical contact. However, if such assessments rely solely on behavioural indicators, they can mask the experience of an animal who may be immobilised yet still suffering from pain. This is particularly the case for processes that induce paralysis, such as electro-immobilisation, where motor responses are suppressed without necessarily resulting in unconsciousness (EFSA, 2004; Gräns et al., 2016; Kestin, Wotton & Adams, 1995).

The European Food Safety Authority (EFSA) defines unconsciousness as a “state of unawareness (loss of consciousness) in which there is temporary or permanent damage to brain function and the individual is unable to perceive external stimuli (which is referred to as insensibility) and control its voluntary mobility and, therefore, respond to normal stimuli, including pain” (EFSA, 2004, p. 16).

Under this definition, insensibility can be a component or consequence of unconsciousness, but it does not guarantee it. Thus, considering that an animal may be sensible to pain until the point of unconsciousness, effective stunning must induce unconsciousness immediately to avoid pain or suffering from the stunning method itself (EFSA, 2004; Humane Slaughter Association (HSA), 2016; World Organisation for Animal Health (WOAH), 2015).

This distinction is critical as the interval between the initiation of a stun and the onset of unconsciousness may expose fish to substantial suffering, particularly where the method involves aversive stimuli such as air exposure, crowding or physical trauma. Therefore, the speed at which unconsciousness is achieved should be considered a critical indicator when assessing the welfare impact of a stunning method.

#### Accuracy and reliability, and the risk of mis-stuns

The accuracy and reliability of a stunning method have critical welfare implications for fish, as an inaccurate or unreliable stun can mean a fish may have to undergo repeated or prolonged stuns, or undergo the killing method whilst still conscious (Wahltinez et al., 2024). Some stunning methods, such as percussive or in-air electrical stunning, rely on targeted application, accurate parameters, and sufficient force for the stun to be effective (Hjelmstedt et al., 2024; Retter et al., 2018; Sundell, Brijs & Gräns, 2024). Inaccuracy in this context can result in considerable welfare impacts on the fish, and may be influenced by several factors, including fish size, equipment parameters, operator skill, diligence, and fish orientation (Espmark et al., 2025; Hjelmstedt et al., 2024; Sundell, Brijs & Gräns, 2024; Van De Vis et al., 2020).

Beyond outright failures, prolonged induction latency (time to unconsciousness) in potentially noxious methods (*e.g.*, electrical) creates a period during which conscious fish are exposed to the stimulus. For example, if total exposure is 20 s but loss of consciousness is not achieved until ∼5 s, the fish experiences those initial seconds while still fully sensible. Because this latency is rarely apparent at the outfeed of a stunner, the immediacy of induction should be evaluated explicitly and separately from maintenance of unconsciousness: first, verify that unconsciousness is induced within a very short exposure window (*e.g.*, <0.5 s or <1 s) using appropriate indicators; only then should longer exposure durations be calibrated to ensure unconsciousness is maintained until death (Sundell, Brijs & Gräns, 2024).

A “mis-stun”, in this framework, refers to any stunning attempt that fails to immediately induce unconsciousness, whether due to inadequate parameters, poor precision, or species/size effects, thereby exposing the fish to experiencing pain and distress. Mis-stuns are not typically reported or recorded and can go undetected if appropriate consciousness indicators are not used (Bowman et al., 2020; Van De Vis et al., 2020). Given the significant potential for welfare compromise, both immediacy of induction and reliability should be treated as critical, separately validated performance criteria when evaluating the effectiveness of stunning methods.

#### Recovery risk and welfare impacts of stun reversibility

Stunning is often a reversible process unless it is paired with an immediate and effective killing method or delivered with sufficient intensity and duration to be lethal. Where loss of consciousness is only transient, and the fish regains consciousness before or during the killing step, the animal is again susceptible to pain, both from any physical trauma caused by the stunning attempt and from the killing procedure itself (Brijs et al., 2021; Espmark et al., 2025). Consequently, methods with a high recovery risk, or a short window of unconsciousness, whether inherent to the method or as a result of operational inconsistencies, variable stunning parameters, or species-specific challenges, represent a significant welfare concern (Brijs et al., 2021; Espmark et al., 2025; Sundell, Brijs & Gräns, 2024; Van De Vis et al., 2020). Furthermore, delays in applying the adjunctive killing method also pose a considerable risk (Hjelmstedt et al., 2022), so the stunning method must facilitate a prompt and reliable killing procedure. Otherwise, a previously stunned but now sentient animal may be subjected to the killing processes while capable of suffering (Mercogliano et al., 2024).

#### Conflicting findings between indicators of stunning success

Given the inherent risk of mis-stuns with all stunning methods, operators must assess individual fish for signs of consciousness to prevent unnecessary pain and suffering. In commercial settings, animal-based measures (ABMs) such as the return of equilibrium (righting reflex), the vestibulo-ocular reflex (eye-roll reflexes, VOR), opercular movements, and responses to stimuli are commonly used as practical indicators that require no specialised equipment (Clemente et al., 2023; Espmark et al., 2025; Gräns et al., 2016; Gräns et al., 2025; Hjelmstedt et al., 2025).

Whilst practical, ABMs vary in reliability across species and contexts. For instance, some ABMs can persist after a fish has lost consciousness, or cease whilst they are still conscious, creating scope for error and impaired welfare (Bowman et al., 2020; Brijs et al., 2025; Brijs et al., 2021; Clemente et al., 2023; Gräns et al., 2016; Hjelmstedt et al., 2025). This can be compounded by human error and subjectivity. The lack of standardisation or species-specific validation of ABMs in aquaculture further undermines both welfare protection in practice and comparability across studies (Bowman et al., 2020; Humane Slaughter Association (HSA), 2018).

Comparatively, electroencephalograms (EEGs) provide direct measures of brain activity (Hjelmstedt et al., 2024; Rucinque et al., 2023; Sundell, Brijs & Gräns, 2024) and, when paired with the measurement of visually evoked responses (VERs), offer robust evidence of loss of consciousness (Hjelmstedt et al., 2022). This typically involves measuring brain activity in response to a visual cue, usually a light which is repeatedly presented at regular intervals. VERs are one of the final functions to be lost before the brain can no longer process information from the environment and are therefore regarded as reliable and robust indicators of brain failure and unconsciousness (Bowman et al., 2020; Hjelmstedt et al., 2022; Kestin, Wotton & Gregory, 1991).

To date, EEG assessments have not been considered feasible in a commercial setting to date, and much of the evidence base has therefore come from non-commercial scale studies with limited external validity (Sundell, Brijs & Gräns, 2024). Recent technical and methodological advances, however, are beginning to bridge this gap, with emerging studies now conducting EEG investigations on fish farms (*e.g.*, Hjelmstedt et al., 2024). In the meantime, ABMs are currently more readily relied upon in practice, making their limitations a critical welfare concern. For instance, given their susceptibility to misinterpretation, ABMs may be better regarded as proxies for welfare compromise, rather than definitive indicators of stunning success (Gräns et al., 2016; Hjelmstedt et al., 2024; Sundell, Brijs & Gräns, 2024). Therefore, the conflict between ABM reliability and EEG accuracy highlights both a gap in current practice and a key welfare factor for evaluating stunning methods.

## Evaluating and Scoring Approach

To evaluate the welfare impacts of different stunning methods used in aquaculture, we assessed each stunning method against the key welfare factors identified in ‘Key Factors Influencing Welfare at Stunning’. To identify relevant literature, we conducted structured searches for each method using Google Scholar for publications between January 2015 and July 2025. The literature review on stunning methods was restricted to studies published from January 2015 onwards to ensure relevance to current commercial practices, technologies, and regulatory contexts, as stunning methods and their implementation have evolved substantially over the last few decades. We used combinations of keywords related to each stunning technique and the welfare-related themes derived from ‘Key Factors Influencing Welfare at Stunning’ (see Supplementary Material S1 for the full search strings).

To qualify for inclusion, studies had to report on the welfare impacts of the stunning method in question in farmed fish, either through direct indicators of consciousness (*e.g.*, EEG, ABMs) or welfare-relevant outcomes (*e.g.*, injury, aversion, recovery time). Whilst consideration was given to the commercial relevance of the study’s methodology, to maximise the evidence base, all studies, regardless of whether they took place at commercial scale or not, were included. Citations were annotated to provide contextual detail.

For each method, the welfare factors were assessed against (a) the likelihood of the welfare factor occurring in practice (*e.g.*, how likely is air-exposure, crowding, or mis-stuns?); (b) the welfare impact should it occur; and (c) the strength of the relevant evidence available. Likelihood was ‘High’ when a welfare factor was consistently reported as relevant across the available literature, ‘Medium’ when reported occasionally or inconsistently, and ‘Low’ or ‘Not applicable’ when rarely or never reported. Because the volume of the literature differs considerably between methods, a simple count of references would have unfairly biased against less-studied methods. Instead, likelihood was assessed proportionally within the available evidence for each method, with consistency weighted more heavily than quantity. For example, if two out of three available studies in a given area identified the welfare factor as relevant, this was considered sufficient to classify it as ‘High’. While this approach involves some interpretative judgement, it provides a more equitable basis for comparing methods with uneven levels of research.

A similar approach was taken to assess the welfare impacts if a given factor occurred. Impact was judged according to the intensity of behavioural and physiological responses to the stressor, the likely resulting affective state, and the duration or reversibility of the effect (see Table 1).

**Table 1:** Definitions and examples for welfare impact scoring categories in stunning and killing fish in aquaculture.

**Impact level**	**Description**	**Example**
High	Intense suffering, pain and/or fear	Air exposure; behavioural aversion
Medium	Measurable stress and/or discomfort; transient	Physical injuries (bruising, scale loss) if rendered unconscious soon after (within minutes)
Low	Minimal or negligible welfare consequences	Physical trauma, if immediately rendered unconscious

To provide transparency about the strength and volume of the underlying evidence, we recorded the amount of literature available on each welfare factor. The following scale was used to accommodate the relatively small body of literature for some areas: 0 studies, 1 study, 2-3 separate studies, and 4 or more separate studies. The results were also annotated with ‘multiple spp.’, ‘few spp.’ or ‘1 sp.’ where appropriate, to indicate the breadth of species represented. Furthermore, full citations and details are provided in the supplementary materials (S2–S6). As mentioned, the likelihood assessment was not based on the volume of evidence, and the evidence score did not override this. Instead, it was included to provide transparency about the strength of the knowledge base underlying the welfare impact conclusions and to highlight the critical absence of research in some areas.

## Evaluation of Stunning Methods

This section evaluates the main stunning methods currently used in commercial aquaculture against the welfare considerations outlined above. A combination of text and comparative tables is used to synthesise the evidence and present the critical welfare issues for each method. For all methods, the species involved, the commercial usage, and any relevant regulations and codes pertaining to their use are summarised in Table 2.

**Table 2:** Overview of main stunning methods used in commercial aquaculture, including the species to which they are applied, the extent of commercial usage, and relevant regulatory or code of practice guidance.

**Stunning method**	**Species (in EU aquaculture)**	**Commercial usage**	**Regulations/Codes *etc***
In-air-(dry/semi-dry) electrical stunning	Salmon (multiple species, *Salmo spp.*), trout (multiple species, *Oncorhynchus spp.*), Arctic char (*Salvelinus alpinus*), European seabass (*Dicentrarchus labrax*), gilthead sea bream (*Sparus aurata*), European eel (*Anguilla anguilla*), pangasius (*Pangasianodon hypophthalmus*), turbot (*Scophthalmus maximus*), barramundi (*Lates calcarifer*), yellowtail kingfish (*Seriola lalandi*), African sharptooth catfish (*Clarias gariepinus*), channel catfish (*Ictalurus punctatus*), claresse (*Clarias spp.*×*Pangasius spp.* hybrids), pike perch (*Sander lucioperca*), and cleaner fish (various wrasse species, *Labridae*).	Commercially employed in larger systems, especially for species like salmon and sea bream.	The WOAH Aquatic Animal Health Code states that “In semi-dry electrical stunning systems, fish should enter the device head first to ensure rapid and efficient stunning.” (World Organisation for Animal Health (WOAH), 2015). Any stunning method, including dry electrical stunning, must be validated and monitored to ensure it meets these welfare standards (World Organisation for Animal Health (WOAH), 2015). EFSA states that the method often leads to delayed or inconsistent loss of consciousness, depending on contact quality, species, and skin condition, and has expressed a preference for in-water electrical stunning (EFSA, 2009c).
In-water electrical stunning	Atlantic salmon, rainbow trout, European seabass, gilthead seabream, tilapia (*Oreochromis spp.*), catfish (*Ictalurus spp.* and *Pangasius spp.*).	Suitable for small to medium-sized fish, and for large numbers (World Organisation for Animal Health (WOAH), 2015).	The WOAH Aquatic Animal Health Code states that the fish must remain fully submerged in the water, and the electrical current within the stunning tank or chamber should be evenly distributed (World Organisation for Animal Health (WOAH), 2015). Likewise, the European Food Safety Authority (EFSA) provides species-specific opinions on stunning parameters and welfare indicators, which guide best practice without legal force (EFSA, 2009b; European Commission, 2017).
Carbon dioxide (CO_2_) stunning	Atlantic salmon, rainbow trout, European eel, carp (*Cyprinus carpio*), and tilapia. Unsuitable for air-breathing species.	CO_2_ stunning has declined greatly in usage, particularly in Europe, where it is considered an inhumane method and is largely being phased out (Bowman et al., 2020).	EFSA and WOAH both caution that CO_2_ stunning can be aversive, causing distress before loss of consciousness and is therefore not considered humane (EFSA, 2004; World Organisation for Animal Health (WOAH), 2015). Likewise, the Aquaculture Stewardship Council (ASC) updated its farm standards in November 2024, stating that CO_2_ baths are prohibited. Instead, fish must be stunned humanely, with alternatives which render fish unconscious instantly (ASC International, 2024). It is also banned in some countries, such as Norway, where it is banned by definition due to loss of consciousness not being immediate (Espmark et al., 2025).
Percussive stunning	Atlantic salmon, rainbow trout, African sharptooth catfish, occasionally tilapia, carp, and pangasius.	Considered labour-intensive and impractical for farms that slaughter large numbers of fish within a short timeframe (Sundell, Brijs & Gräns, 2024).	EFSA and WOAH Aquatic Animal Health Code recognise percussive stunning as an acceptable method if it causes immediate unconsciousness and is followed by a killing step that prevents recovery (EFSA, 2009b; World Organisation for Animal Health (WOAH), 2015).
Gas mixtures	Atlantic salmon, rainbow trout, common carp (and other cyprinids), European seabass, gilthead seabream, and European eel. Unsuitable for air-breathing species.	This method is less common than percussive or electrical stunning, and is considered an alternative to CO_2_, primarily to reduce stress and pain for the fish.	Both EFSA and the WOAH Aquatic Animal Health Code emphasise that any gaseous stunning method must reliably induce immediate and sustained unconsciousness and should be followed by a method to ensure death, in order to safeguard fish welfare (EFSA, 2009b; World Organisation for Animal Health (WOAH), 2015).

Unless otherwise stated, the processes involved with each stunning method are subject to slight variation depending on the farm size, layout, number, and species of fish involved. In particular, the time between the pre-stunning phase and the stunning induction may vary depending on the farm’s layout and the systems in place. As a result, there may also be some variation in terms of the relevant welfare factors to consider and the degree of impact on fish. In addition, some fish may be slaughtered on-site. In contrast, others may be transported in from elsewhere, introducing another set of considerations. For this review, we have focused on the immediate processes before stunning and slaughter, which include the movement from the ‘home pen’ (at the slaughter location) to the stunning pen or equivalent. The following assessment is based on what is most typical based on the available evidence. It is also important to note that we have deliberately not included thermal shock in the scope of the study, as we do not consider it a stunning method, but as a killing method in the realm of air asphyxia.

To provide an overview of the evidence base, the findings from the evaluation and scoring are summarised in Table 3 to indicate the strength of the evidence base supporting the scores made. This table is intended as a comparative guide to highlight the key strengths and weaknesses of each stunning method. The detailed evidence behind these scores is presented in both the supplementary materials (S2–S6) and in the following sections.

**Table 3:** Synthesis of reported likelihood, welfare impact, and strength of evidence across each stage of the process (pre-stunning, induction, and loss of consciousness). For gas narcosis methods (CO_2_ and alternative gas mixtures), the term ‘pre-stunning phase’ is used to denote the immediate pre-induction period, and ‘stunning induction’ is used to denote the introduction of the gases to the fish. For the following assessment, a total of 35 articles were used for in-air electrical stunning; 22 for in-water electrical stunning; 17 for carbon dioxide narcosis; 23 for percussive stunning; and 10 for alternative gas mixtures.

**In-Air (Dry or Semi-Dry) Electrical Stunning**			
	**Likelihood**	**Welfare impact**	**Evidence**
**Pre-stunning Phase**			
Crowding	High	High	4+ studies (general)
Handling	Variable	Variable	4+ studies (general)
Air exposure	High	High	4+ studies (general)
**Stunning induction (excluding impacts of mis-stuns)**			
Behavioural aversion	Unknown	Unknown	2-3 studies (few spp.)
Physiological stress response	High	Unknown	2-3 studies (few spp.)
Physical trauma	Medium	Low	1 study
**Loss of consciousness and recovery risk**			
Risk of delayed onset of unconsciousness	Unknown	High	4+ (multiple spp.)
Risk of failed induction of unconsciousness	Medium	High	4+ (multiple spp.)
Likelihood of regaining consciousness before death	High	High	4+ (multiple spp.)
Conflicting findings between behavioural indicators and EEGs	Unknown	High	2–3 studies (few spp.)
**In-water electrical stunning**			
	**Likelihood**	**Welfare impact**	**Evidence**
**Pre-stunning phase**			
Crowding	High	High	4+ studies (general)
Handling	Variable	High	4+ studies (general)
Air exposure	Variable	High	4+ studies (general)
**Stunning induction (excluding impacts of mis-stuns)**			
Behavioural aversion	Low/**N/A**	Potentially no impact	0 studies
Physiological stress response	High	Unknown	4+ studies (few spp.)
Physical trauma	Medium	Unknown	4+ studies (few spp.)
**Loss of consciousness and recovery risk**			
Risk of delayed onset of unconsciousness	Variable	High	4+ (multiple spp.)
Risk of failed induction of unconsciousness	Medium	High	4+ (multiple spp.)
Likelihood of regaining consciousness before death	High	High	4+ (multiple spp.)
Conflicting findings between behavioural indicators and EEGs	High	High	4+ studies (few spp.)
**Carbon dioxide narcosis**			
	**Likelihood**	**Welfare impact**	**Evidence**
**Pre-stunning phase**			
Crowding	High	High	4+ studies (general)
Handling	Variable	High	4+ studies (general)
Air exposure	Variable	High	4+ studies (general)
**Stunning induction**			
Behavioural aversion	High	High	4+ (multiple spp.)
Physiological stress response	High	Unknown	4+ studies (few spp.)
Physical trauma	High	Low - High	4+ studies (general) 0 studies (method)
**Loss of consciousness and recovery risk**			
Risk of delayed onset of unconsciousness	High	High	4+ (multiple spp.)
Risk of failed induction of unconsciousness	High	High	4+ (multiple spp.)
Likelihood of regaining consciousness before death	Unknown	High	1 study
Conflicting findings between behavioural indicators and EEGs	Unknown	High	1 study
**Percussive stunning**			
	**Likelihood**	**Welfare impact**	**Evidence**
**Pre-stunning phase**			
Crowding	High	High	4+ studies (general)
Handling	High	Low - High	4+ studies (general)
Air exposure	High	High	4+ studies (general)
**Stunning induction (excluding impacts of mis-stuns)**			
Behavioural aversion	Unknown	Unknown	0 studies
Physiological stress response	High	Unknown	2–3 studies (1 sp.)
Physical trauma	High	Low	4+ studies (general)
**Loss of consciousness and recovery risk**			
Risk of delayed onset of unconsciousness	Low	High	2–3 studies (few spp.)
Risk of failed induction of unconsciousness	High	High	2–3 studies (few spp.)
Likelihood of regaining consciousness before death	Medium	High	4+ studies (few spp.)
Conflicting findings between behavioural indicators and EEGs	High	High	2–3 studies (few spp.)
**Gas mixture narcosis**			
	**Likelihood**	**Welfare impact**	**Evidence**
**Pre-stunning phase**			
Crowding	High	High	4+ studies (general)
Handling	Variable	High	4+ studies (general)
Air exposure	Variable	High	4+ studies (general)
**Stunning induction**			
Behavioural aversion	High	High	1 study
Physiological stress response	High	Unknown	4+ studies (general) 0 studies (method)
Physical trauma	High	Low - High	4+ studies (general)
**Loss of consciousness and recovery risk**			
Risk of delayed onset of unconsciousness	High	High	1 study
Risk of failed induction of unconsciousness	High	High	1 study
Likelihood of regaining consciousness before death	Unknown	High	0 studies
Conflicting findings between behavioural indicators and EEGs	Unknown	High	4+ studies (general) 0 studies (method)

### In-air (dry or semi-dry) electrical stunning processes

#### Overview of in-air electrical processes

For best practice of in-air (dry and semi-dry) electrical stunning, the fish should enter the stunner in a single layer, head-first, to ensure a consistent current path and reduce the risk of pre-shocks. Orientation may be automated or manual, with fish placed on a conveyor with a conductive base and overhead electrodes (Espmark et al., 2025; Humane Slaughter Association (HSA), 2016; Lambooij et al., 2010). In semi-dry systems, the fish are sprayed with water after de-watering (Humane Slaughter Association (HSA), 2016). A high-voltage current is then passed through the fish’s brain to induce immediate unconsciousness (Espmark et al., 2025).

#### Overall welfare profile of in-air electrical processes

The potential strengths of in-air electrical stunning are that, when used accurately, the stun is immediate in inducing a state of unconsciousness (see Table 2 and S2). This is a valuable strength, as prolonged onset of unconsciousness is a significant welfare concern. Furthermore, at least for larger species, handling can be automated and therefore reduced compared with some methods, making it a practical and efficient method.

However, there are multiple weaknesses of the method, which can introduce notable welfare issues. Fish must still be handled either manually or automatically to orient them for the stunner, and this process can cause stress or physical harm (Brijs et al., 2025; González-Garoz et al., 2025; Van De Vis et al., 2020). The method also requires exposure to air, which, even for a brief time, is known to be extremely aversive and stressful for fish (Schuck-Paim et al., 2025) and may contribute to the elevated physiological stress responses often reported for this method compared with others (Gräns et al., 2016).

In the stunning induction stage, there is a medium risk of physical trauma because of the stun. However, if the stun is immediate and lasts until death, this should not impact the fish’s welfare. According to the literature reviewed, there is also a medium risk of mis-stuns for this method, as the stun’s effectiveness is highly sensitive to fish size, species, orientation, and equipment load (Espmark et al., 2025; Gräns et al., 2016; Van de Vis et al., 2024). This creates a considerable chance of error, meaning that some fish are inadequately stunned and remain conscious with associated welfare concerns (Brijs et al., 2021; Espmark et al., 2025). Further to this, while not reported in laboratory studies, in practice, fish can be observed experiencing pre-shocks when they are about to enter the stunner, showing aversive behavioural responses, such as flapping and struggling, before entering the stunner and being stunned successfully (A Gräns, pers. obs., October, 2025).

Studies also report variable durations of unconsciousness, including significant individual differences within species (Brijs et al., 2025; Daskalova et al., 2016; Gräns et al., 2016; Hjelmstedt et al., 2024; Van de Vis et al., 2024). This is particularly relevant on farms where killing does not immediately follow stunning or the killing method itself is not immediate (*e.g.*, ice slurry, asphyxia or exsanguination), as it creates the risk of fish recovering consciousness.

Lastly, and this welfare factor appeared across methods, there seems to be considerable conflict between behavioural and neurophysiological indicators of consciousness (Daskalova et al., 2016; Hjelmstedt et al., 2024). This can mean that it is not possible to reliably ascertain unconsciousness using the available indicators, and that in a commercial setting, individuals may inadvertently remain conscious and sensible to pain throughout the following stages. This is an area needing considerable further research; regardless of the method, there is a need for indicators to be reliable in detecting any individuals who are still conscious following stunning, so that they may be immediately re-stunned.

Although in-air electrical stunning has been studied across multiple species, underreporting remains in key areas, especially in reporting the frequency of mis-stuns and recovery of consciousness.

### In-water electrical stunning processes

#### Overview of in-water electrical stunning processes

The process for in-water electrical stunning is similar to that of in-air electrical stunning, in that the fish are typically crowded and transferred to the stunning area (Daskalova, 2019; Espmark et al., 2025; Jung-Schroers et al., 2020; Rucinque et al., 2021). Typically, fish are dewatered and moved into a pipe system, through which an electric current passes through the fish, intended to expose their brains to a strong enough current to induce immediate, but reversible unconsciousness if performed correctly (Espmark et al., 2025; Hjelmstedt et al., 2022).

#### Overall welfare profile of in-water electrical stunning

The strengths of in-water electrical stunning are that, in theory, there is no need for the fish to be handled. In the current commercial reality, however, fish are still often both handled and air-exposed in these systems (A Gräns, pers. obs., October, 2025). There are no reports of behavioural aversion to the stun itself, unless it is a mis-stun. This is because when it is performed correctly (laboratory) studies show that it can immediately induce unconsciousness (Brijs et al., 2021; Hjelmstedt et al., 2025; Sundell, Brijs & Gräns, 2024). This is an important strength of this method, as it removes unnecessary stress and pain and meets a key criterion for an effective stun (see ‘Time to unconsciousness’).

However, according to our review, there are multiple welfare concerns associated with the method. For instance, handling and air exposure are still relevant welfare issues for this method, and are known to be aversive for fish (Brijs et al., 2025; González-Garoz et al., 2025; Schuck-Paim et al., 2025; Van De Vis et al., 2020). In the stunning induction stage, electrical stunning results in both higher (Daskalova et al., 2016; Jung-Schroers et al., 2020) and lower (Oliveira Filho et al., 2021; Retter et al., 2018) physiological stress responses than other methods. It is unclear which aspects of the process cause this, meaning further research is needed in this area. With this method, there is also a medium risk of physical trauma from the stun (Dehcheshmeh et al., 2025; Rucinque, Watanabe & Molento, 2018); however, provided that the stun is immediate and lasts until death, this may not impact the fish’s welfare.

Mis-stuns were considered as a medium risk in terms of likelihood, as there were consistent reports in the literature of both fully successful stuns and mis-stuns in multiple species (Brijs et al., 2021; Jung-Schroers et al., 2020; Veit et al., 2017). However, the welfare impact of a mis-stun, should it occur, is high, as they are likely to cause considerable pain and suffering to the fish. The likelihood of a fish regaining consciousness before death was also considered high following multiple reports of short and varied periods of unconsciousness (Brijs et al., 2021; Hjelmstedt et al., 2022; Retter et al., 2018; Sundell, Brijs & Gräns, 2024; Van De Vis et al., 2020; Veit et al., 2017). This level of unreliability and variability is concerning, especially when the killing method does not immediately follow the stun.

Lastly, as with in-air stunning, the typical indicators used in commercial settings may not be accurate in detecting consciousness in individuals, as results from EEGs and VERs differ (Hjelmstedt et al., 2022; Retter et al., 2018). As this appears to be a widespread issue across the methods, further research is urgently needed to ensure that commercial settings have usable and reliable indicators available to them.

The in-water electrical method appears to be the most studied of the stunning methods; however, the number of species featured in these studies is still dwarfed by the range and scale of commercial aquaculture in the EU and globally. Therefore, further research and development are still urgently needed to fully address and understand the welfare implications associated with this method.

### Carbon dioxide

#### Overview of carbon dioxide (CO_2_) narcosis

As with most stunning processes for CO_2_, fish are typically crowded and moved into a stunning tank that is already saturated with CO_2_. The fish then remain in the tank, with CO_2_ being continuously bubbled in, until they are considered unconscious, and removed for exsanguination (EFSA, 2004). The CO_2_creates a hypercapnic environment for the fish, which can be fatal if the fish are left for long enough (EFSA, 2004). However, if they are removed before death, fish can regain consciousness when in air or if placed into well-oxygenised water (EFSA, 2004; Erikson, 2011).

#### Overall welfare profile of carbon dioxide (CO_2_) narcosis

In terms of strengths, fish stunned using CO_2_ do not typically need to be handled or exposed to the air whilst conscious, offering advantages in that regard (see Table 2 and S4). However, there are multiple significant welfare concerns associated with the method, with considerable welfare implications for the fish involved. Firstly, fish exposed to CO_2−_saturated water show extreme aversive behaviour, including violent movements and escape attempts, which can also result in physical trauma, especially when in crowded conditions (EFSA, 2009d; Poli et al., 2005; Van De Vis et al., 2003). This is not surprising, since topical application of CO_2_ excites nociceptors (pain receptors) in rainbow trout (Mettam, McCrohan & Sneddon, 2012) and causes behavioural responses in zebrafish that are prevented by the use of pain-relieving drugs (Lopez-Luna et al., 2017).

As with all stunning methods, there is a physiological stress response to the process, although surprisingly not as high as for other methods, despite the adverse behavioural response (Gräns et al., 2016; Oliveira Filho et al., 2021). Therefore, it remains unclear the welfare impact of this heightened behavioural response, and to what part of the process it is in response to.

The CO_2_ method does not meet the key criterion of inducing an immediate stun, as it can take several minutes for fish to reach unconsciousness, during which time they show marked behavioural signs of distress (Bowman et al., 2020); Gräns et al., 2016; Oliveira Filho et al., 2021; Rucinque, Watanabe & Molento, 2018; Vargas Baldi et al., 2018). Additionally, the time taken to induce unconsciousness varies between individuals (Bowman et al., 2020; Gräns et al., 2016; Oliveira Filho et al., 2021; Rucinque, Watanabe & Molento, 2018; Vargas Baldi et al., 2018), further highlighting the unreliability of this method. There are also reports that fish quickly regain consciousness when removed from the CO_2_-saturated water, and some become conscious during the killing method (EFSA, 2004; Humane Slaughter Association (HSA), 2016). However, in our review period (2015–2025), we only found one study referring to this welfare factor, which found that none of the fish (Arctic char) regained consciousness during the 10 min of observation (Gräns et al., 2016).

Similarly, there was insufficient evidence to determine the likelihood of conflicting findings between behavioural indicators and EEGs. The limited evidence that does exist to date suggests that, as with other methods, there are concerning conflicts between indicators, meaning that unconsciousness may not be attributed reliably (Bowman et al., 2020).

Because carbon dioxide narcosis is generally considered to be highly aversive, it is effectively banned in some countries (see Table 1). However, it is also often considered a practical and less labour-intensive method, so it remains commonly used globally and has consequently received a fair amount of attention in terms of research.

### Percussive stunning

#### Overview of percussive stunning

The pre-stunning processes for percussive stunning are likely to be similar to those of other methods, where the fish are crowded into a pen. For percussive stunning, fish will also be removed from the water and restrained, either by the operator, mechanical arms, or sidewalls that hold the fish tightly in position (EFSA, 2004; Espmark et al., 2025). The percussive stun may be manually performed using a club or hammer or mechanically with a non-penetrative captive bolt (EFSA, 2004; Espmark et al., 2025). The blow is intended to cause a brain haemorrhage or damage that is sufficient to cease brain function (Lambooij et al., 2010). If performed correctly, this stun should be irreversible (Espmark et al., 2025).

#### Overall welfare profile of percussive stunning

Percussive stunning has the potential to induce immediate and irreversible unconsciousness, provided it is delivered with sufficient force and accuracy (see Table 2 and S5) (Espmark et al., 2025; Robb et al., 2000). In principle, this is a considerable welfare advantage, as it avoids both prolonged induction and the risk of recovery.

However, our review found consistent evidence that an effective stun is not routinely achieved (Jung-Schroers et al., 2020; Retter et al., 2018). For instance, mis-stuns are common with this method, creating a high risk that some fish remain conscious after the first blow (Retter et al., 2018). Mishits, where the stroke impacts the skull but fails to reach the brain region, are a common cause of ineffective stunning, as is the use of insufficient force (EFSA, 2009a; Jung-Schroers et al., 2020; Retter et al., 2018). This issue can become more pronounced as fish mature since changes in skull morphology may require greater force (Roth et al., 2007). Furthermore, ensuring an accurate placement with currently available technology is difficult for smaller or variably sized fish, for individuals exhibiting movement or struggle and under conditions of high-throughput processing (Brijs et al., 2021; Gross et al., 2024). Given the injurious nature of percussive stunning, mishits are likely to be painful. Recovery of consciousness has been documented after manual percussive stunning with a club (“priest”) (Brijs et al., 2021; Hjelmstedt et al., 2025), whereas with handheld pneumatic captive-bolt devices, failed stuns have so far been reported primarily in large (8–15 kg) white sturgeon (Brijs et al., 2025; Gross et al., 2024; Hjelmstedt et al., 2022; Sundell, Brijs & Gräns, 2024). Both failed induction and recovery of consciousness pose significant welfare concerns for fish. Similarly, physical trauma is inherent to the method (Sundell, Brijs & Gräns, 2024). However, if the stun is successful, the fish should remain unconscious and insensible to pain.

Handling is generally required for percussive stunning, although automation can reduce but not eliminate it, and its welfare impact may depend on how it is performed. For example, manual handling may be more stressful and harmful for fish due to potential differences in pressure and unpredictability during restraint, compared with the uniformity of mechanical restraining devices (Danylchuk et al., 2008). In addition, fish also have to be removed from the water for percussive stunning, which, even for a short period, is highly aversive for fish (Schuck-Paim et al., 2025).

As with other methods, there is also evidence of conflict between EEGs and behavioural indicators for percussively stunned fish (Hjelmstedt et al., 2025). Further research is therefore needed to develop reliable, practical indicators of unconsciousness for commercial settings.

### Gas mixture narcosis

#### Overview of gas mixture narcosis

The process for using gas mixtures for stunning fish is similar to that used in carbon dioxide narcosis (‘Overview of carbon dioxide (CO_2_) narcosis’). The only difference is the combination of gases used. Different combinations of gases have been tested in terrestrial species and are currently being tested for their welfare impacts in fish, including carbon monoxide (CO) and combinations of inert gases such as nitrogen (N) and argon (Ar) (Espmark et al., 2025).

#### Overall welfare profile of gas mixture narcosis

Unlike some of the other methods, gas mixtures for fish have received very little research attention to date. Consequently, many areas remain grossly understudied, and the likelihood of welfare issues is unknown. For instance, our review only found one study reporting the impact of this method on fish behaviour, which reported highly aversive reactions in seabream to CO_2_+N_2_+O_2_ and CO_2_+N_2_ (Roque et al., 2021). Whilst limited in scope and species, there are indications from elsewhere in the literature to suggest that the aversiveness of this method is widespread (EFSA, 2004; Erikson, 2011; Espmark et al., 2025). It can be assumed that, as a result of the aversive behaviour, fish are also highly likely to experience physical trauma, especially when in crowded conditions. Injuries such as fractures, scale loss, and bruising can occur when fish show violent escape responses (EFSA, 2009d; Poli et al., 2005; Van De Vis et al., 2003).

As with carbon dioxide stunning, the method does not induce an immediate state of unconsciousness in the fish (Roque et al., 2021). The likelihood of fish regaining consciousness is not known due to a lack of research. Whilst this method is under-researched, evidence so far suggests aversiveness to CO_2_+N_2_+O_2_ and CO_2_+N_2_; however, research is extremely limited and may not be prioritised given the severity of welfare concerns demonstrated in the study by Roque et al. (2021).

## Synthesis of welfare profiles

None of the currently available methods included in this review performs sufficiently across all welfare factors (Table 2 and S2–S6). Each method has inherent risks, with the potential for considerable welfare impact. It was outside the scope of this review to assign relative weightings to the welfare factors considered, therefore a ‘high likelihood’ for one welfare factor is not necessarily more important than a ‘high likelihood’ for another. Consequently, this synthesis focuses on method strengths, weaknesses, and consistencies, rather than ranking them.

During the pre-slaughter or induction phase, all methods are likely to involve crowding, handling, and air exposure, with substantial welfare impacts. Furthermore, gas methods also show poor performance on other welfare factors, such as behavioural aversion and time taken to induce unconsciousness, representing some of the most serious welfare concerns across all stunning methods (Lines & Spence, 2012).

Both electrical methods and percussive stunning have the potential to induce unconsciousness rapidly and without behavioural signs of aversion, which is a considerable strength. However, electrical stunning can have inconsistent but sometimes marked physiological stress responses (Daskalova et al., 2016; Gräns et al., 2016; Jung-Schroers et al., 2020; Oliveira Filho et al., 2021; Retter et al., 2018). Further research is needed to determine the welfare impact of these stress responses and to identify the particular stressors involved, including how to reduce them.

Across all methods, there is a medium to high risk of mis-stuns. Mis-stuns represent a serious welfare flaw. Understanding the factors resulting in mis-stuns and developing reliable mitigation strategies should be a research priority in this field. Similarly, the maintenance of unconsciousness is also of critical importance.

Electrical and percussive methods appear vulnerable to species and size effects, and individual variation is a consistent issue for the duration of unconsciousness (Espmark et al., 2025; Hjelmstedt et al., 2024; Van de Vis et al., 2024). This can be a challenge from a commercial perspective as a stronger stun may be more effective in maintaining unconsciousness, but is not always optimal for product quality (Matos et al., 2010; Morzel, Sohier & Van DeVis, 2003; Roth, Slinde & Robb, 2007; Veit et al., 2017). Consequently, electrical methods are associated with poor maintenance of unconsciousness, introducing a serious risk of fish regaining consciousness (Hjelmstedt et al., 2022; Retter et al., 2018; Sundell, Brijs & Gräns, 2024).

Finally, a fundamental issue applicable to all methods is that behavioural indicators were often contradicted by neurophysiological measures such as VERs. Whilst EEGs and VERs may be considered the gold standard for determining unconsciousness, they are currently not practical in commercial settings. In the absence of technological development in portable EEG/VER technology, behavioural or other visual indicators are needed so that operators can reliably assess consciousness and protect the welfare of individual fish. This cross-cutting issue limits confidence in all the methods and the welfare outcomes arising from them. Therefore, further research to validate practical indicators is essential and could support reliable reporting of mis-stuns.

Overall, these findings show that all currently used methods in commercial aquaculture settings involve significant welfare trade-offs. Some methods reduce pre-slaughter stressors but then perform poorly during induction or recovery. In contrast, others have the potential to achieve immediate unconsciousness but then are associated with high risks of mis-stuns or fish regaining consciousness before death. All methods suffer from a lack of validation of practical indicators for consciousness, with inconsistent findings reported across the board. However, despite the welfare challenges, all forms of stunning that demonstrate the potential to induce immediate unconsciousness represent a marked improvement for the future of fish welfare.

Although it is important to note that current evidence suggests that killing methods such as asphyxia and ice slurry generally cause greater welfare risks than the use of stunning, this conclusion depends critically on the frequency of mis-stuns. To this end, unfortunately, comparative quantification remains scarce. When stunning fails, fish may be subjected both to the potentially painful effects of the stunning attempt and then to the subsequent aversive killing method (*e.g.*, ice slurry or asphyxia). In such cases, the combined welfare burden may equal, or even exceed, that of the killing method alone, despite appearing more acceptable from an external perspective. This underlines the need to minimise mis-stuns and to ensure that stunning methods are applied reliably before they can be considered genuine welfare improvements over non-stunning practices.

## Discussion

This review aimed to evaluate the commonly used stunning methods for fish in aquaculture systems against a cohort of welfare factors, highlighting the strengths and weaknesses of each method and identifying areas in need of further research and development. It revealed issues across all stunning methods, including a widespread disparity in results in critical areas such as immediacy of unconsciousness, the risk of mis-stuns, and contradictions regarding reliable, yet practical, indicators of unconsciousness.

### Complexities in applying stunning methods for fish

A core problem underlying these issues is the lack of species-specific provisions and indicators. Even within one species, differences in size can reduce the reliability of the stunning method. Whilst uniformity is desired at slaughter, a mixture of sizes in fish being slaughtered is often inevitable, especially when multiple species are involved (Humane Slaughter Association (HSA), 2018; James et al., 2025; Mercogliano et al., 2024; Sae-Lim et al., 2015). Variations in body size and species-specific physiology can thus influence the effectiveness and welfare outcomes of stunning methods (HSA, 2018; Mercogliano et al., 2024). For example, larger-than-average individuals may not be effectively stunned under electrical stunning, leaving them sensible to pain and suffering, whereas smaller individuals may be irreversibly stunned.

In contrast, percussive stunning may reliably render larger fish unconscious, but smaller individuals are more prone to painful mishits. For some stunning methods, species differences in skin thickness, hypoxia resistance, stress sensitivity, or behavioural responses necessitate species-specific parameters, and may limit their applicability across species (EFSA, 2009a; EFSA, 2009b; EFSA, 2009e). These factors, therefore, add considerable complexity to both the application and assessment of stunning methods in commercial practice. Future work is needed to establish species and size-specific parameters for relevant stunning methods and to validate these findings under commercial-scale conditions, ensuring effective stunning across all species farmed in aquaculture.

Another core problem underlying such comparisons and categorisations is the fact that electrical stunning is not a single “method” but a tightly parameter-dependent process. Small changes in frequency and waveform (AC, DC, pulsed DC, duty cycle, DC offset), voltage/current density, exposure time, field uniformity, electrode geometry and placement, fish orientation and spacing (load), water conductivity/salinity and temperature, as well as species, size, skin/mucus condition and prior stress state, can shift outcomes from immediate unconsciousness to mere electro-immobilisation, or even injury. Because these factors interact non-linearly, broad labels like “in-water electrical” or “dry electrical” obscure the determinants of welfare; generalising across systems risks appearing sound on paper while failing in practical application. Meaningful comparisons, therefore, require full parameter reporting, species- and size-specific validation (ideally with EEG/VER), and treatment of electrical stunning as a tuned process with defined critical control points. The development of robust future standards for any of these methods will also rely heavily on validation specific to all parameters, including both animal-based and protocol-based criteria.

The reliance on non-commercial scale testing of stunning methods has likely contributed to some of the inconsistencies seen both in practice and in research, as many studies fail to replicate the conditions of large-scale commercial systems. Most of the studies in our review were limited to very small sample sizes compared with the scale of aquaculture slaughter in practice. For instance, 19 of the studies included were performed at a research scale. In contrast, only four were on a commercial scale (see supplementary materials S2–S6). This lack of commercially tested research can reduce the relevance of these studies to real-world contexts and complicates the evaluation of stunning methods under practical conditions. Consequently, future research that seeks to validate stunning methods at commercial scales is urgently needed to confirm that welfare outcomes are maintained under practical slaughter conditions.

In addition to welfare outcomes, stunning practices can also influence product quality, including fillet colour, texture, haemorrhaging and shelf life. For instance, prolonged struggling, delayed loss of consciousness, and aversive stunning conditions can impair product quality, as can the use of excessive electrical parameters (Morzel, Sohier & Van DeVis, 2003; Lambooij et al., 2007; Mercogliano et al., 2024; Gräns et al., 2026). However, whilst these effects are important to industry decision-making, a detailed evaluation of product quality impacts was beyond the scope of this review.

The role of the operator is another important factor that may not be detected in research, both in terms of skill and adherence to protocols, as this can significantly influence the success of stunning. Findings from studies on terrestrial farmed animals and the role of the operator at slaughter can provide interesting insights into this understudied area. For example, whilst slaughterhouse workers give high priority to animal welfare, their perception, and ultimately their rating of an animal’s welfare is often higher than that provided by an expert assessor (Lipovšek et al., 2024). Furthermore, attitudes of slaughterhouse workers can vary considerably, as can understanding of relevant legislation, which can impact how the animals are treated (Lipovšek et al., 2024; Pastrana-Camacho, Estévez-Moreno & Miranda-de la Lama, 2023). Therefore, quantifying the effect of operator variability, both in terms of the implementation of stunning protocols and in the utilisation of consciousness indicators, within this context would provide helpful insight for developing and improving training standards and identifying critical control points to ensure consistent welfare outcomes. Further to this, deviations from manufacturer-recommended maintenance procedures have been observed and may contribute to failed stuns; however, the frequency and extent of this problem are not well documented (A Gräns, pers. obs., October 2025). The development of standardised training in operating procedures, as well as in species-specific welfare assessment, could significantly improve the treatment of fish during the stunning and killing process.

### Towards reliable indicators of consciousness

Given the variability introduced by species, size, operator factors, and the methods themselves, reliable and accurate monitoring of consciousness is of utmost importance. Without validated indicators, even the best stunning method cannot guarantee good welfare in practice. Advances in consciousness sensors for terrestrial farmed animals demonstrate the potential for automated systems, which can reduce operator error and fatigue while providing faster and more consistent assessments suitable for high-throughput slaughter lines (Voogt et al., 2023). However, these systems still require training with validated behavioural proxies for unconsciousness, reinforcing the urgent need to develop species-specific indicators for consciousness. Moreover, accurate reporting on the number of mis-stuns can also contribute to the development and refinement of suitable stunning methods.

### Limitations

Despite the growing body of literature evaluating stunning methods in aquaculture, several structural limitations in the current evidence base constrain robust welfare assessment. First, the scope of studies included was constrained by the inherent biases of the literature, with some methods and species underrepresented, in light of the strong research focus on the limited number of commercially dominant species. Whilst we aimed to reflect the weight and context of the evidence behind our scores, greater insight from a wider range of species and contexts may have yielded different conclusions.

Second, while the assessment was guided by a consistent framework, welfare evaluation in this field necessarily involves synthesising studies with differing methodologies, scales and species, and outcome measures, and that at times report conflicting findings. While a transparent framework was used to reduce subjectivity, some degree of interpretation is unavoidable given current methodological heterogeneity.

Third, welfare research on stunning commonly evaluates individual factors in isolation. Consequently, the cumulative and interactive effect of multiple stressors is rarely captured. For example, fish who experience physical trauma during crowding may have a reduced capacity to cope with subsequent processes compared to a fish who has not been injured. Recognising the importance of individuals’ experiences is a critical component of welfare and therefore requires attention in future research.

Finally, the very challenges highlighted in the review, such as the difference in species needs, variations in size, and variability in operator skill and compliance, introduce substantial variability into stunning and slaughter outcomes. These factors complicate the derivation of universal conclusions and limit the appropriateness of weighting welfare factors. Accordingly, rather than producing aggregated scores, this review seeks to identify consistent trends and critical concerns, and to provide a platform for collaborative work with industry and researchers to refine methods, improve indicators, and enhance welfare outcomes for the billions of fish slaughtered each year in aquaculture.

### Recommendations

Based on the findings of this review, it is clear that no single stunning method is currently successful in achieving optimal welfare for fish across all the key welfare factors (‘Evaluation of Stunning Methods’ and Table 3). Therefore, there is a dual need to (a) mandate and support continued research and development into technologies that introduce new methods of stunning or refine current methods of stunning that minimise fish welfare risks, and (b) generate welfare-based criteria to guide both the refinement of existing methods and the development of future technologies. Doing so will aid the establishment of effective procedures for stunning and slaughtering fish to safeguard welfare. The welfare factors used in this review have been translated into a set of welfare-based criteria to guide the evaluation, refinement, and development of stunning methods in the future (Table 4). It is important to note that these criteria represent key welfare goals for stunning methods in aquaculture, intended to provide a conceptual framework to highlight priorities for welfare improvements, rather than validated and measurable criteria. Future work is needed to translate these criteria into measurable parameters, species-specific thresholds, and validated monitoring protocols suitable for commercial aquaculture systems.

**Table 4:** Conceptual welfare-based criteria proposed to guide evaluation and innovation in aquaculture stunning methods. The criteria are framed as overarching welfare goals (robustness, avoidance of injury, adaptability to species/size variation, and commercial feasibility) rather than as validated indicators, to support the development and assessment of stunning approaches. To support the application of these welfare-based criteria, factors such as ease of application, operational costs, and worker safety must also be considered as key requirements to ensure sustainability and high feasibility.

**Phase**	**Welfare-Based Criterion:**
**Pre-stunning**	
Crowding	Minimise crowding during handling and transfer.
Individual handling	Avoid individual handling where possible to reduce stress and injury.
Group handling	Minimise group handling (e.g., brailing/netting) to reduce stress and injury risk.
Air exposure	Avoid air exposure to reduce stress and negative affective states.
**Stunning induction**	
Behavioural aversion	Not elicit any behavioural signs of distress or avoidance.
Physiological stress	Minimise physiological stress responses.
Physical trauma	Avoid causing physical injury, except when required to induce immediate and maintained unconsciousness.
**Loss of consciousness and recovery risk**	
Delayed unconsciousness	Immediately induce unconsciousness.
Failed induction	Reliably render fish unconscious. Procedures in place to deal with failed stuns.
Maintaining unconsciousness	Maintain unconsciousness until death.
Indicator reliability	Allow for an accurate assessment of consciousness using practical indicators.
**Robustness of method**	
Commercial applicability	Robust under commercial-scale conditions.
Species variation	Adaptable to the species being farmed, with demonstrated welfare efficacy for each.
Size variation	Tolerant of individual size variation.

As our review has shown, notable disparities exist between the effectiveness of stunning methods demonstrated under laboratory conditions and their performance under commercial aquaculture settings. This highlights the clear need for future research to be developed under laboratory conditions and subsequently validated in operational environments to ensure relevance, applicability and meaningful impact on industry practices. However, this can only be achieved in tandem with developments in technology, as current methods for stunning are ineffective at a commercial scale. Therefore, without such technological advances, even well-designed field research may fail to translate into consistent, industry-wide improvements.

In addition to technological advancements, there are also operational and policy measures that can be implemented immediately to enhance welfare while new solutions are being developed. Therefore, to meet the criteria outlined in Table 4, a combined focus on applied research, technological innovation, and operational/policy enhancements is critical to bridge the gap between experimental success and real-world impact. Achieving this cannot rely on voluntary action or informal knowledge sharing alone, as competitive pressures and the proprietary nature of technical innovation limit the dissemination of improved practices. Instead, progress is most likely to be achieved through structured public–private partnerships, underpinned by regulatory oversight and supported by targeted financial incentives, which together create the conditions for widespread uptake of welfare-improving solutions. In particular, we recommend the following priority areas for improving fish welfare during stunning.

Technological/Research-focused priorities:

⋅ Investment in technological research and development of new stunning methods that meet the requirements for sustained humane stunning (as outlined in Table 4), prioritising validation under commercial settings.

⋅ Development of scientifically validated and commercially adaptable key animal welfare indicators for use during stunning, which can be applied and monitored on farm.

⋅ Creation of structured operator training schemes to ensure technician proficiency in using welfare indicators, minimising the risk of mis-stuns, and promptly recognising and addressing them when they occur.

⋅ Application of the criteria outlined in Table 4 to guide on-farm investigations aimed at improving the performance and effectiveness of current methods, thereby reducing harm until more effective technologies become available. For example, ensuring fish remain in water at all times during in-water electrical stunning.

Operational and policy-focused priorities (immediate enhancements):

⋅ Introduction of regulation mandating the use of stunning in fish farming and prohibiting methods that are scientifically demonstrated to cause suffering or frequent mis-stuns.

⋅ Provision of financial incentives or subsidies to support the replacement of systems that are scientifically evidenced to induce suffering and show no scope for improvement (*e.g.*, gas stunning).

⋅ Commercial investment in technician capacity and skill development to reduce human error leading to mishandling, mis-stuns, or air-exposure.

⋅ Establishment of monitoring and auditing frameworks to verify compliance, track stunning outcomes and identify welfare bottlenecks, supported by the development of shared industry databases and digital welfare monitoring tools.

Collectively, these recommendations highlight how progress in technology, training, and regulation can close the gap between experimental evidence and commercial practice, ensuring genuine improvements in fish welfare. However, reliance on voluntary action is insufficient; public authorities must actively establish, enforce, and oversee good practice, including through the provision of targeted financial incentives or subsidies to support industry uptake of welfare-improving technologies and procedures.

## Conclusion

This review critically evaluates the main stunning methods currently used in aquaculture against welfare factors associated with the stunning process. The welfare outcomes across the methods assessed vary considerably. While some methods show promise under certain conditions, none of them has achieved consistently high welfare outcomes. Key challenges, including the absence of validated species-specific indicators of unconsciousness, the impact of size and species differences, and the role of the operator and the commercial context, remain barriers to reliably ensuring improved welfare outcomes and warrant further exploration.

The welfare-based criteria recommended in this review are not intended as definitive standards, but as a conceptual framework to support and guide the refinement of existing stunning methods and the development of new technologies. Progress within this sector requires cross-sector collaboration between fish welfare researchers, industry and regulators to better understand current practices, the scale of the issues, and to test improvements under commercial conditions.

In the meantime, while new technologies are being developed and validated, producers can take immediate steps to improve welfare. These include selecting currently available stunning methods that minimise harm, avoiding methods that are known to cause high levels of suffering, and implementing operational protocols to reduce high-risk welfare moments, such as mis-stuns, prolonged air exposure, and handling stress. Proactive management of these factors can significantly reduce welfare risks even before novel technologies are widely adopted.

Ultimately, future improvements in the welfare of fish during stunning and slaughter are most likely to come from innovations that combine technological development with scientific research that identifies validated, species-appropriate welfare indicators, alongside robust commercial-scale application.

## Supplemental Information

10.7717/peerj.21258/supp-1 Supplemental Information 1 Search terms used for the systematic review of the literature Each search began with the ‘All searches’ string, followed by one of the five Methods (1-5), and then a welfare factor search string (e.g., ‘crowding’, ‘handling’, etc.).

10.7717/peerj.21258/supp-2 Supplemental Information 2 Detailed welfare assessment for In-Air (Dry or Semi-Dry) Electrical Stunning in aquaculture Reports likelihood, welfare impact and strength of relevant evidence across pre-stunning, induction, and loss of consciousness phases, along with details of the relevant evidence to support the synthesis presented in Table 2.

10.7717/peerj.21258/supp-3 Supplemental Information 3 Detailed welfare assessment for In-Water Electrical Stunning in aquaculture Reports likelihood, welfare impact and strength of relevant evidence across pre-stunning, induction, and loss of consciousness phases, along with details of the relevant evidence to support the synthesis presented in Table 2.

10.7717/peerj.21258/supp-4 Supplemental Information 4 Detailed welfare assessment for carbon dioxide narcosis in aquaculture Reports likelihood, welfare impact and strength of relevant evidence across pre-stunning, induction, and loss of consciousness phases, along with details of the relevant evidence to support the synthesis presented in Table 2.

10.7717/peerj.21258/supp-5 Supplemental Information 5 Detailed welfare assessment for percussive stunning in aquaculture Reports likelihood, welfare impact and strength of relevant evidence across pre-stunning, induction, and loss of consciousness phases, along with details of the relevant evidence to support the synthesis presented in Table 2.

10.7717/peerj.21258/supp-6 Supplemental Information 6 Detailed welfare assessment for Gas Mixture Narcosis in aquaculture Reports likelihood, welfare impact and strength of relevant evidence across pre-stunning, induction, and loss of consciousness phases, along with details of the relevant evidence to support the synthesis presented in Table 2.

## Additional Information and Declarations

### Data Availability

The following information was supplied regarding data availability: This is a literature review.
