# Supplementary material for: Stunning methods in aquaculture slaughter and their implications for fish welfare
Source: PeerJ. 2026 May 18;14:e21258. doi: 10.7717/peerj.21258 (PMC13192462; doi:10.7717/peerj.21258)
Supplement: Supplemental Information 1 — Each search began with the ‘All searches’ string, followed by one of the five Methods (1-5), and then a welfare factor search string (e.g., ‘crowding’, ‘handling’, etc.). [file peerj-14-21258-s001.docx]

S1. Search terms used for the systematic review of the literature. Each search began with the ‘All searches’ string, followed by one of the five Methods (1-5), and then a welfare factor search string (e.g., ‘crowding’, ‘handling’, etc.).

| **All** | |
| --- | --- |
| *All searches* | Stunning OR Slaughter AND fish AND Aquaculture AND… |
| *Methods 1* | Dry electrical OR Semi dry electrical OR In-air electrical AND… |
| *Methods 2* | In-water electrical OR Wet electrical OR Electronarcosis AND… |
| *Methods 3* | Carbon dioxide OR CO_2_ AND… |
| *Methods 4* | Gas mixture OR Gas OR Gases AND… |
| *Methods 5* | Percussive OR Non-penetrative OR Captive-bolt AND… |
| **Pre-stunning** | |
| *Crowding* | Crowding |
| *Handling* | Handling OR Restraint |
| *Air exposure* | Air exposure OR Hypoxia |
| **Stunning induction** | |
| *Behavioural aversion* | Aversion OR Aversive OR Avoidance OR Escape OR Struggling |
| *Physiological stress response* | Stress OR Physiological stress OR Biochemical Stress OR Stress biomarkers OR Cortisol |
| *Physical trauma* | Physical trauma OR Injury OR Injuries OR Lesion OR Fracture OR Pain |
| **Loss of consciousness and reversibility** | |
| *Time to unconsciousness* | Time to unconsciousness OR Onset of unconsciousness OR Latency to unconsciousness |
| *Failed induction* | Failed induction OR Mis-stuns OR Inaccurate OR Accurate OR Successful induction OR Successfully inducted |
| *Risk of recovery* | Risk of recovery OR Reversible OR Regained consciousness OR Recover consciousness OR Consciousness recovery OR Remained unconscious OR Irreversible OR Irreversibly stunned |
| *Indicators of consciousness* | Indicators of consciousness OR Signs of consciousness OR Conscious state OR Brain activity OR EEG OR Righting reflex OR Return of equilibrium OR Opercular rate OR Opercular movement OR Spontaneous movement OR VERs OR Behavioural indicators |
