# Supplementary material for: Stunning methods in aquaculture slaughter and their implications for fish welfare
Source: PeerJ. 2026 May 18;14:e21258. doi: 10.7717/peerj.21258 (PMC13192462; doi:10.7717/peerj.21258)
Supplement: Supplemental Information 2 — Reports likelihood, welfare impact and strength of relevant evidence across pre-stunning, induction, and loss of consciousness phases, along with details of the relevant evidence to support the synthesis presented in Table 2. [file peerj-14-21258-s002.docx]

S2: Detailed welfare assessment for In-Air (Dry or Semi-Dry) Electrical Stunning in aquaculture. Reports likelihood, welfare impact and strength of relevant evidence across pre-stunning, induction, and loss of consciousness phases, along with details of the relevant evidence to support the synthesis presented in Table 2.

Key: ∞ Indicates a reference to grey literature; ^µ^ indicates a study or part of a study performed at laboratory/ research scale; and ^α^ indicates a study or part of a study performed at commercial scale.

| **In-Air (Dry or Semi-Dry) Electrical Stunning** | | |
| --- | --- | --- |
| **Pre-Stunning Phase:** | | |
| **Crowding** | | |
| **Likelihood** | **Welfare impact** | **Strength of evidence** |
| High | High | 4+ studies (general) |
| **Relevant evidence** | | |
| Likelihood:  Crowding is a typical pre-stunning process, with varying degrees and duration (Daskalova, 2019; Espmark et al., 2025; Jung-Schroers et al., 2020; Rucinque et al., 2021). Welfare impact:  Not studied explicitly in relation to this method, but the welfare impacts are well documented for multiple species of farmed fish (see section 3.1.1). Relevant evidence:  Because of the risk of physical injury and mortality, the focus has tended to be on the impact on flesh quality, rather than the welfare of fish themselves (Lines and Spence, 2012; Stien et al., 2024). Sub-surface risks include the accidental creation of pockets when nets are tightened, trapping the fish and intensifying crowding issues (Stien et al., 2024). Technology is increasingly being used to address these issues, including the use of winch cameras or remotely operated underwater vehicles to detect sub-surface issues (Stien et al., 2024), acoustic telemetry to monitor fish behaviour (Føre et al., 2018), and stunning devices, such as in-water pipeline electrical systems that can reduce or eliminate the need for pre-stunning crowding (Welfarm, 2023). However, further research is often needed to ascertain the degree to which these methods effectively reduce stress and other welfare impacts in fish. | | |
| **Handling** | | |
| **Likelihood** | **Welfare impact** | **Strength of evidence** |
| Variable | Variable | 4+ studies (general) |
| **Relevant evidence** | | |
| Likelihood:  Fish may be handled to orient them into the right position for the stunner in some systems, but automated positioning is also common (see section 3.1.2) (Brijs et al., 2025; González-Garoz et al., 2025). Welfare impact:  Not studied explicitly in relation to this method, but the welfare impacts are well documented for multiple species of farmed fish (see section 3.1.2). Secondary welfare impacts may derive from the fish being reportedly easier to handle (Gräns et al., 2016; Hjelmstedt et al., 2025), meaning the efficiency and efficacy of throat slitting is improved, which could have a secondary welfare benefit (Gräns et al., 2016).  Relevant evidence:  Whilst the negative impacts of handling are relatively well documented, especially in regard to flesh quality (Matos et al., 2010), they are often overlooked in the assessment of stunning systems themselves, or are grouped with crowding and other pre-slaughter stressors (Brijs et al., 2018; Matos et al., 2010). Whilst a holistic approach ensures all aspects of the stunning process are considered, there is also a need to break down the welfare implications of individual handling methods, to not only ensure that farmers can make evidence-based decisions on the most welfare-friendly options, but also to ensure targeted improvements. For instance, the development and use of rubberised nets can effectively reduce scale loss in fish (Powell, 2021).  Whilst the long-term impacts of scale loss are not relevant for fish bound for slaughter, the short-term impacts, including pain and discomfort, are highly relevant to individual fish and may also negatively impact their ability to cope with additional stressors (Lange et al., 2018; Oliveira and Galhardo, 2009; Petitjean et al., 2019). ‘Fish-friendly’ pumping systems are also under development to reduce shear force and crowding, minimise pressure fluctuations, and avoid impacts and abrasions (Krakers et al., 2015; Pan et al., 2022). However, these systems vary widely in design and effectiveness and lack validation across species. Further research and regulation are therefore needed to ensure meaningful welfare gains from the development of these technologies. | | |
| **Air Exposure** | | |
| **Likelihood** | **Welfare impact** | **Strength of evidence** |
| High | High | 4+ studies (general) |
| **Relevant evidence** | | |

Likelihood:

An inherent part of the method

Welfare impact:

Welfare impacts of air exposure are well documented for multiple species of farmed fish (see section 3.1.3). Welfare impact is worsened with increasing duration of air exposure, but even a brief exposure is considered a significant welfare impact (Schuck-Paim et al., 2025).

Relevant evidence:

Whilst there is little regulatory protection for fish, best practice guidelines and certification schemes generally advise against killing fish by asphyxiation, and whilst some guidelines just state that time out of water should be minimised (European Commission, 2020; WOAH, 2015), others are more detailed. For instance, 15 seconds is increasingly being used as a limit for farmed fish, based on the behavioural responses of some fish becoming more pronounced after 15 seconds (HSA, 2016; RSPCA, 2024, 2020). However, given that there can be considerable variation between species and individuals in terms of responses to stressors, coping abilities, and coping styles, focusing solely on behavioural signs may be too limited (Castanheira et al., 2017; Erikson et al., 2016; Martins et al., 2012). Therefore, given the evidence that fish suffer severe negative affects when exposed to air for a brief time (Schuck-Paim et al., 2025), further research is urgently needed to determine humane thresholds and alternatives to current practices, including the use of stunning methods where fish are not removed from the water.

| **Stunning Induction (excluding impacts of mis-stuns)** | | |
| --- | --- | --- |
| **Behavioural aversion** | | |
| **Likelihood** | **Welfare impact** | **Strength of evidence** |
| Unknown | Unknown | 2-3 studies (few spp.) |
| **Relevant evidence** | | |

Likelihood:

Fish may respond aversively to the air exposure, and so it is unclear if the stun itself is aversive.

Welfare impact:

There are welfare impacts from the air exposure, but it is not clear whether the stun itself is aversive.

Relevant evidence:

No evidence of aversion to an accurate stun (see section 3.2.1); No escape behaviours seen in Arctic char (Gräns et al., 2016)^α^. Although failed semi-dry stunning in catfish results in powerful movements and escape attempts (Hjelmstedt et al., 2024)∞ µ.

| **Physiological stress response** | | |
| --- | --- | --- |
| **Likelihood** | **Welfare impact** | **Strength of evidence** |
| High | Unknown | 2-3 studies (few spp.) |
| **Relevant evidence** | | |

Likelihood:

Based on the limited findings available, all stunning methods result in some degree of physiological stress response (see section 3.2.2), but electrical methods appear to cause a greater response.

Welfare impact:

It is difficult to separate the stress response from pre-stunning stressors from the stunning induction. However, one key stressor, air exposure, is an inherent part of the method, which may result in a higher welfare impact (see sections 3.1.3 and 3.2.2).

Relevant evidence:

Measures of physiological stress, such as cortisol levels, can help to quantifiably assess how fish respond to different slaughter practices (Ellis et al., 2012).
Dry-electrical stunning resulted in elevated plasma cortisol levels in rainbow trout, compared with those stunned by in-water electrical and percussive stunning (Jung-Schroers et al., 2020)∞ µ. Similarly, Arctic char (*Salvelinus alpinus*) who had been dry-stunned had significantly higher plasma cortisol levels, compared with control fish and those stunned with CO_2_ (Gräns et al., 2016)^α^.

| **Physical trauma** | | |
| --- | --- | --- |
| **Likelihood** | **Welfare impact** | **Strength of evidence** |
| Medium | Low | 1 study |
| **Relevant evidence** | | |

Likelihood:

Potentially from the equipment or incorrect settings (see section 3.2.3).

Welfare impact:

If the fish is stunned immediately and remains unconscious until death, then there is likely no welfare impact (see section 3.2.3).
Relevant evidence:

Injuries are typically unwanted from a product quality perspective, and so there is a high motivation to mitigate them.

If the field strength is too high or the frequency is too low, it can cause injuries, including fractures, although the welfare impact of these may not be applicable if the fish is still rendered unconscious (Gräns et al., 2016). However, there can be a secondary welfare impact, as the strength of the electrical stun may be reduced to lower the physical impact, which may mean it is not sufficient to induce an effective stun.

In common carp (*Cyprinus carpio*), farmed fish experienced external injuries as a result of contact with the electrodes (Retter et al., 2018)^α^.

| **Loss of Consciousness and Recovery Risk** | | |
| --- | --- | --- |
| **Risk of delayed onset of unconsciousness** | | |
| **Likelihood** | **Welfare impact** | **Strength of evidence** |
| Unknown | High | 4+ studies (multiple spp.) |
| **Relevant evidence** | | |

Likelihood:

Whilst there is laboratory evidence that providing correct parameters and fittings for species/individual (see section 3.3.1), the risk of delayed onset of unconsciousness is low, observations of these stunners in practice highlight clear issues with fish experiencing pre-shocks before being rendered unconscious (A Gräns, personal observation, October, 2025).

Welfare impact:

If a delay in the onset of unconsciousness occurs, then the welfare impact may be high, as the stunning method can cause physical trauma and pain in fish (see sections 3.2.3 and 3.3.1).

Relevant evidence:

EEG evidence of stun rendering fish immediately unconscious is available for multiple species: Atlantic salmon (van de Vis et al., 2023)^∞^, turbot (*Scophthalmus maximus* )and common sole (*Solea solea*) (Daskalova et al., 2016a)µ, lumpfish (*Cyclopterus lumpus*) (van de Vis et al., 2024)∞ µ. and channel catfish (*Ictalurus punctatus*) (Hjelmstedt et al., 2024)∞ µ.
Behavioural evidence of immediate stun in Arctic char (Gräns et al., 2016).

| **Risk of failed induction of unconsciousness** | | |
| --- | --- | --- |
| **Likelihood** | **Welfare impact** | **Strength of evidence** |
| Medium | High | 4+ studies (multiple spp.) |
| **Relevant evidence** | | |

Likelihood:

Likelihood is subject to correct usage, parameters and fittings for species/individual (see section 3.3.2). Evidence of mis-stuns exists for some species.

Welfare impact:

The welfare impact of a failed stun is considerable if it occurs, as the stunning method can cause physical trauma and pain in fish (see section 3.3.2).
Relevant evidence:
Known risk for dry stunning is that if the stunner becomes overloaded with a double layer of fish on the conveyor belt, then each fish only receives half of the intended voltage, resulting in an insufficient stun (Espmark et al., 2025)^∞^.

Correct electrode placement is noted as critical for ensuring a lasting stun (Hjelmstedt et al., 2024)∞ µ. Muscle contractions can mean the stun needs to be at least 1s to be effective (van de Vis et al., 2024)∞ µ.

Evidence of mis-stuns in common sole and turbot: 74-80% of turbot remained unconscious following the 20s stun, and 80-87% of the sole when subsequently chilled in ice water. Some fish continued to show breathing and respond to taps for up to 75m (Daskalova et al., 2016a)µ.

| **Likelihood of regaining consciousness before death** | | |
| --- | --- | --- |
| **Likelihood** | **Welfare impact** | **Strength of evidence** |
| High | High | 4+ studies (multiple spp.) |
| **Relevant evidence** | | |

Likelihood:

Highly variable across species and parameters.

Welfare impact:

The welfare impact of a fish regaining consciousness following a stun is considerable if it occurs, as the stunning method can cause physical trauma and pain in fish, and the fish may be subjected to slaughter whilst conscious (see sections 3.2.3 and 3.3.3).

Relevant evidence:

Arctic char recovered equilibrium within 4-7 minutes following stunning (Gräns et al., 2016)^α^.

Sole and turbot are thought to remain unconscious for at least 5 minutes following stunning. However, one turbot regained consciousness at around 2min and then lost it again at 3min, indicating transient unconsciousness (Daskalova et al., 2016a)µ.
Lumpfish stunned for 5s and then immersed in CO_2_-saturated water can regain consciousness after 420s (van de Vis et al., 2024)∞ µ.
Catfish stunned for 1s using semi-dry electrical stunning remain unconscious for 10-40s, increasing to 45 – 240s when stunned for 6s; however, if electrode placement was inaccurate, this reduced to 0-100s (Hjelmstedt et al., 2024)∞ µ.

| **Conflicting findings between behavioural indicators and EEGs** | | |
| --- | --- | --- |
| **Likelihood** | **Welfare impact** | **Strength of evidence** |
| Unknown | High | 2-3 studies (few spp.) |
| **Relevant evidence** | | |

Likelihood:

There is insufficient evidence to conclude the likelihood.

Welfare impact:

There is a significant welfare impact if unconsciousness is inaccurately assessed (see section 3.3.4).
Relevant evidence:

EEG readings reported unconsciousness in turbot and sole, whilst both species showed continued behavioural signs of consciousness, including responding to noxious stimuli such as tapping, and light breathing (Daskalova et al., 2016a)µ.

In catfish, the presence of visually evoked responses (VERs) correlated with visible behavioural signs of consciousness (Hjelmstedt et al., 2024)∞ µ.
