# Supplementary material for: Stunning methods in aquaculture slaughter and their implications for fish welfare
Source: PeerJ. 2026 May 18;14:e21258. doi: 10.7717/peerj.21258 (PMC13192462; doi:10.7717/peerj.21258)
Supplement: Supplemental Information 3 — Reports likelihood, welfare impact and strength of relevant evidence across pre-stunning, induction, and loss of consciousness phases, along with details of the relevant evidence to support the synthesis presented in Table 2. [file peerj-14-21258-s003.docx]

S3: Detailed welfare assessment for In-Water Electrical Stunning in aquaculture. Reports likelihood, welfare impact and strength of relevant evidence across pre-stunning, induction, and loss of consciousness phases, along with details of the relevant evidence to support the synthesis presented in Table 2.
Key: ∞ Indicates a reference to grey literature; ^µ^ indicates a study or part of a study performed at laboratory/ research scale; and ^α^ indicates a study or part of a study performed at commercial scale.

| **In-Water Electrical Stunning** | | |
| --- | --- | --- |
| **Pre-Stunning Phase:** | | |
| **Crowding** | | |
| **Likelihood** | **Welfare impact** | **Strength of evidence** |
| High | High | 4+ studies (general) |
| **Relevant evidence** | | |

Likelihood:

Crowding is a typical pre-stunning process, with varying degrees and duration (Daskalova, 2019; Espmark et al., 2025; Jung-Schroers et al., 2020; Rucinque et al., 2021). However, the development of in-water pipeline electrical systems promises to remove the need for crowding (Welfarm, 2023) ^∞^.

Welfare impact:

Not studied explicitly in relation to this method, but the welfare impacts are well documented for multiple species of farmed fish (see section 3.1.1).
Relevant evidence:
Degree and duration of crowding can vary from farm to farm (Jung-Schroers et al., 2020).
Because of the risk of physical injury and mortality, the focus has tended to be on the impact on flesh quality, rather than the welfare of fish themselves (Lines and Spence, 2012; Stien et al., 2024). Sub-surface risks include the accidental creation of pockets when nets are tightened, trapping the fish and intensifying crowding issues (Stien et al., 2024). Technology is increasingly being used to address these issues, including the use of winch cameras or remotely operated underwater vehicles to detect sub-surface issues (Stien et al., 2024), acoustic telemetry to monitor fish behaviour (Føre et al., 2018), and stunning devices, such as in-water pipeline electrical systems that can reduce or eliminate the need for pre-stunning crowding (Welfarm, 2023). However, further research is often needed to ascertain the degree to which these methods effectively reduce stress, and other welfare impacts in fish.

| **Handling** | | |
| --- | --- | --- |
| **Likelihood** | **Welfare impact** | **Strength of evidence** |
| Variable | High | 4+ studies (general) |
| **Relevant evidence** | | |

Likelihood:

In most systems, fish are typically handled in order to be de-watered before being placed into the new controlled water. However, whilst this occurs regularly in practice, in theory it should not be required.

Welfare impact:

Not studied explicitly in relation to this method, but the welfare impacts are well documented for multiple species of farmed fish (see section 3.1.2).
Relevant evidence:

Whilst the negative impacts of handling are relatively well documented, especially in regard to flesh quality (Matos et al., 2010), they are often overlooked in the assessment of stunning systems themselves, or are grouped with crowding and other pre-slaughter stressors (Brijs et al., 2018; Matos et al., 2010). Whilst a holistic approach ensures all aspects of the stunning process are considered, there is also a need to break down the welfare implications of individual handling methods, to not only ensure that farmers can make evidence-based decisions on the most welfare-friendly options, but also to ensure targeted improvements. For instance, the development and use of rubberised nets can effectively reduce scale loss in fish (Powell, 2021).
Whilst the long-term impacts of scale loss are not relevant for fish bound for slaughter, the short-term impacts, including pain and discomfort, are highly relevant to individual fish and may also negatively impact their ability to cope with additional stressors (Lange et al., 2018; Oliveira and Galhardo, 2009; Petitjean et al., 2019). ‘Fish-friendly’ pumping systems are also under development to reduce shear force and crowding, minimise pressure fluctuations, and avoid impacts and abrasions (Krakers et al., 2015; Pan et al., 2022). However, these systems vary widely in design and effectiveness and lack validation across species. Further research and regulation are therefore needed to ensure meaningful welfare gains from the development of these technologies.

| **Air exposure** | | |
| --- | --- | --- |
| **Likelihood** | **Welfare impact** | **Strength of evidence** |
| High | High | 4+ studies (general) |
| **Relevant evidence** | | |

Likelihood:

In many systems, fish are first de-watered and exposed to air before going into the new controlled water. However, whilst this occurs regularly in practice, in theory it should not be required.

Welfare impact:

Welfare impacts of air exposure are well documented for multiple species of farmed fish (see section 3.1.3). Welfare impact is worsened with increasing duration of air exposure, but even a brief exposure is considered a significant welfare impact (Schuck-Paim et al., 2025).

Relevant evidence:

Whilst there is little regulatory protection for fish, best practice guidelines and certification schemes generally advise against killing fish by asphyxiation, and whilst some guidelines just state that time out of water should be minimised (European Commission, 2020; WOAH, 2015), others are more detailed. For instance, 15 seconds is increasingly being used as a limit for farmed fish, based on the behavioural responses of some fish becoming more pronounced after 15 seconds (HSA, 2016; RSPCA, 2024, 2020). However, given that there can be considerable variation between species and individuals in terms of responses to stressors, coping abilities, and coping styles, focusing solely on behavioural signs may be too limited (Castanheira et al., 2017; Erikson et al., 2016; Martins et al., 2012). Therefore, given the evidence that fish suffer severe negative affects when exposed to air for a brief time (Schuck-Paim et al., 2025), further research is urgently needed to determine humane thresholds and alternatives to current practices, including the use of stunning methods where fish are not removed from the water.

| **Stunning Induction (excluding impacts of mis-stuns)** | | |
| --- | --- | --- |
| **Behavioural aversion** | | |
| **Likelihood** | **Welfare impact** | **Strength of evidence** |
| Low | Not applicable | 0 studies |
| **Relevant evidence** | | |

Likelihood:

No evidence to suggest the stunning induction is aversive for fish.

Welfare impact:

No evidence of aversion to an accurate stun.
Relevant evidence:
No evidence of aversion to an accurate stun.

| **Physiological stress response** | | |
| --- | --- | --- |
| **Likelihood** | **Welfare impact** | **Strength of evidence** |
| High | Unknown | 4+ studies (few spp.) |
| **Relevant evidence** | | |

Likelihood:

Based on the limited findings available, all stunning methods result in some degree of physiological stress response, but electrical methods appear to cause a greater response.

Welfare impact:

Unclear and sometimes mixed findings: It is difficult to separate the stress response from pre-stunning stressors from the stunning induction (see section 3.2.2), and findings are mixed even within species.
Relevant evidence:

Evidence of both greater and lower physiological stress responses in comparison to other methods.

Mixed findings for common carp; cortisol levels are both significantly higher (Daskalova et al., 2016b)^µ^ and lower (Retter et al., 2018)^α^ in electrically stunned carp compared to those stunned by percussion. Cortisol levels are significantly lower in electrically stunned fish than those left to asphyxiate (Daskalova et al., 2016b)^µ^.

In pacu (*Piaractus mesopotamicus*), electrically stunned fish had non-significantly lower cortisol levels than those stunned with CO_2_ (Oliveira Filho et al., 2021)^µ^.

Rainbow trout (*Oncorhynchus mykiss*) electrically stunned, followed by percussion, results in significantly higher cortisol levels compared to trout stunned by manual percussion or just electrically stunned (Jung-Schroers et al., 2020)^α^ µ.

| **Physical trauma** | | |
| --- | --- | --- |
| **Likelihood** | **Welfare impact** | **Strength of evidence** |
| Medium | Unknown | 4+ studies (few spp.) |
| **Relevant evidence** | | |

Likelihood:

There is a medium risk of physical harm due to the equipment or incorrect settings being used (see section 3.2.3).

Welfare impact:

If the fish is stunned immediately and remains unconscious until death, then there is likely no welfare impact, but if not, the welfare impact will be high (see section 3.2.3).
Relevant evidence:

High electrical fields, current densities, low frequencies and prolonged exposures are known to cause injuries in fish, including fractures and haemorrhages (Hjelmstedt et al., 2025). However, such injuries are typically discussed in relation to product quality.

No fractures detected in pacu (Rucinque et al., 2018)^µ^, or in binni (*Mesopotamichthys sharpeyi*) (Dehcheshmeh et al., 2025)^µ^. Nor were any gill tissue damage detected in binni (Dehcheshmeh et al., 2025)^µ^.

| **Loss of Consciousness and Recovery Risk** | | |
| --- | --- | --- |
| **Risk of delayed onset of unconsciousness** | | |
| **Likelihood** | **Welfare impact** | **Strength of evidence** |
| Variable | High | 4+ studies (general) |
| **Relevant evidence** | | |

Likelihood:

Providing correct parameters and fittings for the species/individual, the likelihood should be low. However, there are mixed findings across species, so the likelihood of occurrence is variable.

Welfare impact:

The welfare impact of a failed stun is considerable if it occurs, as the stunning method can cause physical trauma and pain in fish (see section 3.3.2).
Relevant evidence:
EEG evidence that stun is immediate in multiple species: Nile tilapia (*Oreochromis niloticus*) (Sundell et al., 2024)^µ^, channel catfish (*Ictalurus punctatus)* (Hjelmstedt et al., 2025)^µ^, rainbow trout (Hjelmstedt et al., 2022)^µ^, sharptooth catfish (*Clarias gariepinus*) (Brijs et al., 2021)^µ^, and lumpfish (van de Vis et al., 2024)∞ µ.
Behavioural evidence that stun is immediate in juvenile salmonids (Bouwsema et al., 2022)^µ^ and rainbow trout (Saraiva et al., 2024)^µ^.
Mixed findings for pacu, with behavioural evidence of immediate stun in one study (Rucinque et al., 2018)^µ^, and non-immediate (~30s) in another (Oliveira Filho et al., 2021)^µ^.
Mixed results with common carp; In one study, 23.1% of the sample showed injuries resulting from mishits, and therefore were not stunned immediately (Retter et al., 2018)^µ^, similar reports from other studies (Daskalova et al., 2016b)^µ.^

| **Risk of failed induction of unconsciousness** | | |
| --- | --- | --- |
| **Likelihood** | **Welfare impact** | **Strength of evidence** |
| Medium | High | 4+ studies (general) |
| **Relevant evidence** | | |

Likelihood:

There are mixed reports and evidence regarding the occurrence of failed induction of consciousness.

Welfare impact:

The welfare impact of a fish regaining consciousness following a stun is considerable if it occurs, as the stunning method can cause physical trauma and pain in fish, and the fish may be subjected to slaughter whilst conscious (see sections 3.2.3 and 3.3.3)
Relevant evidence:
Reliability of the stun is influenced by: how well the stunner is maintained, as changes in salinity over time will alter the electrical field and reduce the stunner's reliability; whether proper use is abided by; and whether the maximum number of fish being stunned at any time is in line with manufacturer guidance (Espmark et al., 2025)^∞^.
Poor reliability is seen in rainbow trout in practice; only 2/10 and 14/20 trout from the farm sample showed behavioural signs of unconsciousness following electrical stunning (Jung-Schroers et al., 2020)^α.^
Silver catfish and rainbow trout stunned for 1s showed immediate aversive behavioural responses indicating a failure to stun (Hjelmstedt et al., 2022; Veit et al., 2017)µ.
All rainbow trout stunned for 20s were immediately rendered unconscious (Saraiva et al., 2024)^µ^.
Sharptooth catfish stunned for 2s experienced an immediate epileptic like seizure but showed visually evoked responses (VERs) upon its cessation (Brijs et al., 2021)^µ^. A 5s stun had variable results across individuals, and a 10s stun meant a loss of VERs in all fish post-stun (Brijs et al., 2021)^µ^. Juvenile salmonids were considered to be immediately rendered unconscious (according to behavioural indicators) after a 20-second stun (Bouwsema et al., 2022)^µ^.

| **Likelihood of regaining consciousness before death** | | |
| --- | --- | --- |
| **Likelihood** | **Welfare impact** | **Strength of evidence** |
| High | High | 4+ studies (general) |
| **Relevant evidence** | | |

Likelihood:

Highly variable across species and parameters.

Welfare impact:

The welfare impact of a fish regaining consciousness following a stun is considerable if it occurs, as the stunning method can cause physical trauma and pain in fish, and the fish may be subjected to slaughter whilst conscious (see sections 3.2.3., and 3.3.3.).
Relevant evidence:
Highly variable results depending on consciousness indicators, electrical parameters, and farm processes.
In rainbow trout, electrical stunning has varied effects on the duration of loss of VERs (10-415s), both as a result of parameters and between individuals exposed to the same parameters (Hjelmstedt et al., 2022)^µ^. VERs were transient and often disappeared within <1min (Hjelmstedt et al., 2022)^µ^.
In Nile tilapia, stunning for 1s with low and high intensity resulted in loss of VERs for only ~16s and 66-151s, respectively. Stunning for 30s meant no recovery of VERs for the entire 30-minute observation period (Sundell et al., 2024)^µ^.
In channel catfish, recovery time ranged from ~10s to over 16 minutes (Hjelmstedt et al., 2025).^µ^
In sharptooth catfish, recovery of VERs was immediate for 2s stuns, 0-~2min for 5s stun, and ~3.3min for the 10s stun (Brijs et al., 2021)^µ^.
Common carp stunned for either 1s or 5s showed VERs within 30s post-stunning (Retter et al., 2018)^µ^.
Lumpfish stunned for 10s and had their throats cut, can still recover 7min afterwards (van de Vis et al., 2024)∞.
Silver catfish (*Rhamdia quelen*) stunned for 5s recovered response to painful stimuli and vestibulo-ocular reflex (VOR) after 8.5-57.3s (Veit et al., 2017).
Juvenile salmonids remained unconscious for at least 17 min after stunning (according to behavioural indicators) (Bouwsema et al., 2022)^µ^.
Rainbow trout regained consciousness (according to behavioural indicators) at ~230s (Saraiva et al., 2024)^µ^.

| **Conflicting findings between behavioural indicators and EEGs** | | |
| --- | --- | --- |
| **Likelihood** | **Welfare impact** | **Strength of evidence** |
| High | High | 4+ studies (few spp.) |
| **Relevant evidence** | | |

Likelihood:
Further research is needed as there are mixed findings.

Welfare impact:

There is a significant welfare impact if unconsciousness is inaccurately assessed (see section 3.3.4).
Relevant evidence:
Most research shows a difference between the timing of behavioural and neurophysiological indicators of unconsciousness.
Common carp show VERs following stunning despite showing no behavioural signs of consciousness, rendering them inaccurate for determining unconsciousness (Retter et al., 2018) ^µ^.
Visual indicators such as ventilation took longer to resume in stunned rainbow trout than the return of VERs, indicating that the fish were responsive to their environment before visual signs of consciousness could be detected (Hjelmstedt et al., 2022) ^µ^.
In rainbow trout, breathing movements and VORs were lost at approximately the same time as VERs (Jung-Schroers et al., 2020)µ.
