# Supplementary material for: Stunning methods in aquaculture slaughter and their implications for fish welfare
Source: PeerJ. 2026 May 18;14:e21258. doi: 10.7717/peerj.21258 (PMC13192462; doi:10.7717/peerj.21258)
Supplement: Supplemental Information 4 — Reports likelihood, welfare impact and strength of relevant evidence across pre-stunning, induction, and loss of consciousness phases, along with details of the relevant evidence to support the synthesis presented in Table 2. [file peerj-14-21258-s004.docx]

S4: Detailed welfare assessment for carbon dioxide narcosis in aquaculture. Reports likelihood, welfare impact and strength of relevant evidence across pre-stunning, induction, and loss of consciousness phases, along with details of the relevant evidence to support the synthesis presented in Table 2.
Key: ∞ Indicates a reference to grey literature; ^µ^ indicates a study or part of a study performed at laboratory/ research scale; and ^α^ indicates a study or part of a study performed at commercial scale.

| **Carbon Dioxide Narcosis** | | |
| --- | --- | --- |
| **Pre-Stunning Phase:** | | |
| **Crowding** | | |
| **Likelihood** | **Welfare impact** | **Strength of evidence** |
| High | High | 4+ studies (general) |
| **Relevant evidence** | | |

Likelihood:

Crowding is a typical pre-slaughter process, with varying degrees and duration (Daskalova, 2019; Espmark et al., 2025; Jung-Schroers et al., 2020; Rucinque et al., 2021).

Welfare impact:

Not studied explicitly in relation to this method, but the welfare impacts are well documented for multiple species of farmed fish (see section 3.1.1).

Relevant evidence:

Degree and duration of crowding can vary from farm to farm (Jung-Schroers et al., 2020).
Because of the risk of physical injury and mortality, the focus has tended to be on the impact on flesh quality, rather than the welfare of fish themselves (Lines and Spence, 2012; Stien et al., 2024). Sub-surface risks include the accidental creation of pockets when nets are tightened, trapping the fish and intensifying crowding issues (Stien et al., 2024). Technology is increasingly being used to address these issues, including the use of winch cameras or remotely operated underwater vehicles to detect sub-surface issues (Stien et al., 2024), acoustic telemetry to monitor fish behaviour (Føre et al., 2018), and stunning devices, such as in-water pipeline electrical systems that can reduce or eliminate the need for pre-stunning crowding (Welfarm, 2023). However, further research is often needed to ascertain the degree to which these methods effectively reduce stress, and other welfare impacts in fish.

| **Handling** | | |
| --- | --- | --- |
| **Likelihood** | **Welfare impact** | **Strength of evidence** |
| Variable | High | 4+ studies (general) |
| **Relevant evidence** | | |

Likelihood:

In most systems, fish are typically handled in order to be de-watered before being placed into the new controlled water. However, whilst this occurs regularly in practice, in theory it should not be required.

Welfare impact:

Not studied explicitly in relation to this method, but the welfare impacts are well documented for multiple species of farmed fish (see section 3.1.2).
Relevant evidence:

Whilst the negative impacts of handling are relatively well documented, especially in regard to flesh quality (Matos et al., 2010), they are often overlooked in the assessment of stunning systems themselves, or are grouped with crowding and other pre-slaughter stressors (Brijs et al., 2018; Matos et al., 2010). Whilst a holistic approach ensures all aspects of the stunning process are considered, there is also a need to break down the welfare implications of individual handling methods, to not only ensure that farmers can make evidence-based decisions on the most welfare-friendly options, but also to ensure targeted improvements. For instance, the development and use of rubberised nets can effectively reduce scale loss in fish (Powell, 2021).
Whilst the long-term impacts of scale loss are not relevant for fish bound for slaughter, the short-term impacts, including pain and discomfort, are highly relevant to individual fish and may also negatively impact their ability to cope with additional stressors (Lange et al., 2018; Oliveira and Galhardo, 2009; Petitjean et al., 2019). ‘Fish-friendly’ pumping systems are also under development to reduce shear force and crowding, minimise pressure fluctuations, and avoid impacts and abrasions (Krakers et al., 2015; Pan et al., 2022). However, these systems vary widely in design and effectiveness and lack validation across species. Further research and regulation are therefore needed to ensure meaningful welfare gains from the development of these technologies.

| **Air exposure** | | |
| --- | --- | --- |
| **Likelihood** | **Welfare impact** | **Strength of evidence** |
| Variable | High | 4+ studies (general) |
| **Relevant evidence** | | |

Likelihood:

In many systems, fish are first de-watered and exposed to air before going into the new controlled water. However, whilst this occurs regularly in practice, in theory it should not be required.

Welfare impact:

Welfare impacts of air exposure are well documented for multiple species of farmed fish (see section 3.1.3). Welfare impact is worsened with increasing duration of air exposure, but even a brief exposure is considered a significant welfare impact (Schuck-Paim et al., 2025).

Relevant evidence:

Whilst there is little regulatory protection for fish, best practice guidelines and certification schemes generally advise against killing fish by asphyxiation, and whilst some guidelines just state that time out of water should be minimised (European Commission, 2020; WOAH, 2015), others are more detailed. For instance, 15 seconds is increasingly being used as a limit for farmed fish, based on the behavioural responses of some fish becoming more pronounced after 15 seconds (HSA, 2016; RSPCA, 2024, 2020). However, given that there can be considerable variation between species and individuals in terms of responses to stressors, coping abilities, and coping styles, focusing solely on behavioural signs may be too limited (Castanheira et al., 2017; Erikson et al., 2016; Martins et al., 2012). Therefore, given the evidence that fish suffer severe negative affects when exposed to air for a brief time (Schuck-Paim et al., 2025), further research is urgently needed to determine humane thresholds and alternatives to current practices, including the use of stunning methods where fish are not removed from the water.

| **Induction** | | |
| --- | --- | --- |
| **Behavioural aversion** | | |
| **Likelihood** | **Welfare impact** | **Strength of evidence** |
| High | High | 4+ studies (multiple spp.) |
| **Relevant evidence** | | |

Likelihood:

The method is known to induce extreme aversion in a range of fish species.

Welfare impact:

The display of highly aversive behaviour is indicative of distress in the fish (see section 3.2.1).

Relevant evidence:

Rapid, violent, aversive struggling, surface breathing, and escape responses have been documented in multiple species for considerable periods of time.

Arctic char (several mins) (Gräns et al., 2016)^α^;

Cobia (*Rachycentron canadum*) (31 mins) (Vargas Baldi et al., 2018)^µ^.

Atlantic Halibut (*Hippoglossus hippoglossus*) (<305s) (Rucinque et al., 2023)^µ^.

Pacu (15-25min) (Oliveira Filho et al., 2021)^µ^.

Rainbow trout (>30s) (Bowman et al., 2020)^α^.

| **Physiological stress response** | | |
| --- | --- | --- |
| **Likelihood** | **Welfare impact** | **Strength of evidence** |
| High | Unknown | 4+ studies (few spp.) |
| **Relevant evidence** | | |

Likelihood:

The likelihood is high based on the limited findings available, as all methods result in some degree of physiological stress response.

Welfare impact:

It is difficult to separate the stress response from pre-slaughter stressors from the narcosis.

Relevant evidence:

Despite considerable behavioural signs of aversion, the physiological stress response can be lower than in electrical methods:

In Arctic char, plasma cortisol levels were significantly higher than control fish, but significantly lower than dry-electrical stunned fish (Gräns et al., 2016)^α^.

In pacu, cortisol levels were non-significantly lower in those exposed to CO_2_ than those electrically stunned (Oliveira Filho et al., 2021)^µ^.

| **Physical trauma** | | |
| --- | --- | --- |
| **Likelihood** | **Welfare impact** | **Strength of evidence** |
| High | Low-High | 4+ studies (general)  0 studies (method) |
| **Relevant evidence** | | |

Likelihood:

Physical trauma is considered highly likely as a result of the extreme aversive behaviours performed, which may be escalated if in crowded conditions.

Welfare impact:

The welfare impact varies depending on the scale of the trauma and the duration the fish is conscious for (see section 3.2.3).

Relevant evidence:

No specific evidence was found in the literature in relation to the method, but aversive struggling behaviours can result in physical injuries.

| **Loss of Consciousness and Recovery Risk** | | |
| --- | --- | --- |
| **Risk of delayed onset of unconsciousness** | | |
| **Likelihood** | **Welfare impact** | **Strength of evidence** |
| High | High | 4+ studies (multiple spp.) |
| **Relevant evidence** | | |

Likelihood:

Multiple examples of unconsciousness not being immediate.

Welfare impact:

The welfare impact of delayed unconsciousness is significant as the fish show extreme aversion before unconsciousness (see sections 3.2.1 and 3.3.1).

Relevant evidence:

Considerable evidence shows that CO_2_ narcosis is not immediate and causes prolonged suffering until unconsciousness:
Arctic char; 2-4mins to lose equilibrium (Gräns et al., 2016)^α^.

Cobia; at least 48 minutes to unconsciousness (Vargas Baldi et al., 2018)^µ^

Halibut; 258.8±46.2s to unconsciousness (Rucinque et al., 2023)^µ^

Pacu; ~54mins to unconsciousness (Oliveira Filho et al., 2021)^µ^

Rainbow trout; 3:30-7:99min to lose VERs (Bowman et al., 2020)^α^.

| **Risk of failed induction of unconsciousness** | | |
| --- | --- | --- |
| **Likelihood** | **Welfare impact** | **Strength of evidence** |
| High | High | 4+ studies (multiple spp.) |
| **Relevant evidence** | | |

Likelihood:

Unconsciousness is not immediate with CO_2_ narcosis, and so is considered a ‘failed induction’.

Welfare impact:

The welfare impact of failed unconsciousness is significant as the fish show extreme aversion before unconsciousness (see sections 3.2.1 and 3.3.2).

Relevant evidence:

Considerable evidence shows that loss of consciousness is not immediate for all individuals and that some individuals take longer than expected to become unconscious.

In Nile tilapia, CO_2_ narcosis induced the loss of vestibulo-ocular reflex (VOR) and dorsal fin erection in 95% of individuals within 10 minutes (Camargo-dos-Santos et al., 2021).

| **Likelihood of regaining consciousness before death** | | |
| --- | --- | --- |
| **Likelihood** | **Welfare impact** | **Strength of evidence** |
| Unknown | High | 1 study |
| **Relevant evidence** | | |

Likelihood:

This area is understudied, and the likelihood depends on how long the fish are kept in the water (see section 5.3.2). There are reports of fish regaining consciousness when out of the water (EFSA, 2004; HSA, 2016).

Welfare impact:

The welfare impact of a fish regaining consciousness following narcosis can be considerable, as they are then conscious during the following slaughter processes (see section 3.3.3).

Relevant evidence:

Dependent on the duration of narcosis, the method is not immediate, and so some individuals may be killed during the process, whereas others may not have reached unconsciousness.

Arctic char did not recover from CO_2_ narcosis (10min exposure) (Gräns et al., 2016)^α^.

| **Conflicting findings between behavioural indicators and EEGs** | | |
| --- | --- | --- |
| **Likelihood** | **Welfare impact** | **Strength of evidence** |
| Unknown | High | 1 study |
| **Relevant evidence** | | |

Likelihood:

Limited research in this area has been conducted to determine the likelihood.

Welfare impact:

There is a significant welfare impact if unconsciousness is inaccurately assessed (see section 3.3.4).

Relevant evidence:

Known to be highly contradictory for other methods, but limited evidence in this context.
Rainbow trout remained conscious, according to VERs, up to 3.5 minutes after ventilation was lost and up to 6.5 minutes after the fish lost equilibrium (Bowman et al., 2020)^α^.
