# Supplementary material for: Stunning methods in aquaculture slaughter and their implications for fish welfare
Source: PeerJ. 2026 May 18;14:e21258. doi: 10.7717/peerj.21258 (PMC13192462; doi:10.7717/peerj.21258)
Supplement: Supplemental Information 5 — Reports likelihood, welfare impact and strength of relevant evidence across pre-stunning, induction, and loss of consciousness phases, along with details of the relevant evidence to support the synthesis presented in Table 2. [file peerj-14-21258-s005.docx]

S5: Detailed welfare assessment for percussive stunning in aquaculture. Reports likelihood, welfare impact and strength of relevant evidence across pre-stunning, induction, and loss of consciousness phases, along with details of the relevant evidence to support the synthesis presented in Table 2.

Key: ∞ Indicates a reference to grey literature; ^µ^ indicates a study or part of a study performed at laboratory/ research scale; and ^α^ indicates a study or part of a study performed at commercial scale.

| **Percussive Stunning** | | |
| --- | --- | --- |
| **Pre-Stunning Phase:** | | |
| **Crowding** | | |
| **Likelihood** | **Welfare impact** | **Strength of evidence** |
| High | High | 4+ studies (general) |
| **Relevant evidence** | | |

Likelihood:

Crowding is a typical pre-stunning process, with varying degrees and duration (Daskalova, 2019; Espmark et al., 2025; Jung-Schroers et al., 2020; Rucinque et al., 2021).

Welfare impact:

Not studied explicitly in relation to this method, but the welfare impacts are well documented for multiple species of farmed fish (see section 3.1.1).

Relevant evidence:

Degree and duration of crowding can vary from farm to farm (Jung-Schroers et al., 2020).
Because of the risk of physical injury and mortality, the focus has tended to be on the impact on flesh quality, rather than the welfare of fish themselves (Lines and Spence, 2012; Stien et al., 2024). Sub-surface risks include the accidental creation of pockets when nets are tightened, trapping the fish and intensifying crowding issues (Stien et al., 2024). Technology is increasingly being used to address these issues, including the use of winch cameras or remotely operated underwater vehicles to detect sub-surface issues (Stien et al., 2024), acoustic telemetry to monitor fish behaviour (Føre et al., 2018), and stunning devices, such as in-water pipeline electrical systems that can reduce or eliminate the need for pre-stunning crowding (Welfarm, 2023). However, further research is often needed to ascertain the degree to which these methods effectively reduce stress, and other welfare impacts in fish.

| **Handling** | | |
| --- | --- | --- |
| **Likelihood** | **Welfare impact** | **Strength of evidence** |
| High | Low-High | 4+ studies (general) |
| **Relevant evidence** | | |

Likelihood:

Handling is an inherent part of the method.

Welfare impact:

The welfare impact depends on the methods used (see sections 3.1.2 and 5.4.2)

Relevant evidence:

Handling and restraint are inherent parts of the method, and are known to induce stress in fish (Hjelmstedt et al., 2025; Sundell et al., 2024). However, the welfare impacts are directly associated with the methods used, as mechanical methods may be less invasive (Espmark et al., 2025).

Little specific evidence available, but channel catfish are known to struggle vigorously when handled prior to stunning (Hjelmstedt et al., 2025)^µ^.

| **Air exposure** | | |
| --- | --- | --- |
| **Likelihood** | **Welfare impact** | **Strength of evidence** |
| High | High | 4+ studies (general) |
| **Relevant evidence** | | |

Likelihood:

An inherent part of the method

Welfare impact:

Welfare impacts of air exposure are well documented for multiple species of farmed fish (see section 3.1.3). Welfare impact is worsened with increasing duration of air exposure, but even a brief exposure is considered a significant welfare impact (Schuck-Paim et al., 2025).

Relevant evidence:

Whilst there is little regulatory protection for fish, best practice guidelines and certification schemes generally advise against killing fish by asphyxiation, and whilst some guidelines just state that time out of water should be minimised (European Commission, 2020; WOAH, 2015), others are more detailed. For instance, 15 seconds is increasingly being used as a limit for farmed fish, based on the behavioural responses of some fish becoming more pronounced after 15 seconds (HSA, 2016; RSPCA, 2024, 2020). However, given that there can be considerable variation between species and individuals in terms of responses to stressors, coping abilities, and coping styles, focusing solely on behavioural signs may be too limited (Castanheira et al., 2017; Erikson et al., 2016; Martins et al., 2012). Therefore, given the evidence that fish suffer severe negative affects when exposed to air for a brief time (Schuck-Paim et al., 2025), further research is urgently needed to determine humane thresholds and alternatives to current practices, including the use of stunning methods where fish are not removed from the water.

| **Stunning Induction (excluding impacts of mis-stuns)** | | |
| --- | --- | --- |
| **Behavioural aversion** | | |
| **Likelihood** | **Welfare impact** | **Strength of evidence** |
| Unknown | Unknown | 0 studies |
| **Relevant evidence** | | |

Likelihood:

Fish may respond aversively to the air-exposure and handling, and so it is unclear if the stun itself is aversive (see sections 3.1.2 and 3.1.3).

Welfare impact:

There are welfare impacts from the air exposure and handling, but it is not clear whether the stun itself is aversive.

Relevant evidence:

Inherent parts of the method (air exposure and handling) will likely cause behavioural aversion. However, it is unclear whether the percussion itself causes behavioural aversion unless it is a mis-stun.

| **Physiological stress response** | | |
| --- | --- | --- |
| **Likelihood** | **Welfare impact** | **Strength of evidence** |
| High | Unknown | 2-3 studies (1 sp.) |
| **Relevant evidence** | | |

Likelihood:

All stunning methods incur some degree of physiological stress.

Welfare impact:

Unclear and sometimes mixed findings: It is difficult to separate the stress response from pre-stunning stressors from the stunning induction (see section 3.2.2), and findings are mixed even within species.

Relevant evidence:

Common carp stunned by percussion had significantly elevated mean cortisol levels compared with carp electrically stunned (Retter et al., 2018)^α^.

Common carp stunned by percussion had lower mean cortisol levels than fish stunned by electrical stunning or left to asphyxiate (Daskalova et al., 2016b)^µ.^

| **Physical trauma** | | |
| --- | --- | --- |
| **Likelihood** | **Welfare impact** | **Strength of evidence** |
| High | Low | 4+ studies (general) |
| **Relevant evidence** | | |

Likelihood:

Physical trauma is an inherent part of the method.

Welfare impact:

The welfare impact is low, providing the fish is immediately stunned and does not regain consciousness.

Relevant evidence:

Fish can incur injuries if percussive stunning is performed manually and ineffectively (Sundell et al., 2024).

In common carp, 23.1% of the study sample showed injuries resulting from mishits (Retter et al., 2018)^µ^.

| **Loss of Consciousness and Recovery Risk** | | |
| --- | --- | --- |
| **Risk of delayed onset of unconsciousness** | | |
| **Likelihood** | **Welfare impact** | **Strength of evidence** |
| Low | High | 2-3 studies (few spp.) |
| **Relevant evidence** | | |

Likelihood:

The likelihood is low, provided sufficient force and accuracy are used.

Welfare impact:

The welfare impact of a failed stun is considerable if it occurs, as the stunning method can cause physical trauma and pain in fish (see section 3.3.2).

Relevant evidence:

Evidence of mis-stuns where unconsciousness is not immediate:
Rainbow trout; 11/141 showed behavioural signs of consciousness following stunning (Jung-Schroers et al., 2020)^α.^

Common carp; 23.1% of the study sample showed injuries resulting from mishits (Retter et al., 2018)^µ^.

| **Risk of failed induction of unconsciousness** | | |
| --- | --- | --- |
| **Likelihood** | **Welfare impact** | **Strength of evidence** |
| High | High | 2-3 studies (few spp.) |
| **Relevant evidence** | | |

Likelihood:

High proportions of mis-stuns are reported in studies.

Welfare impact:

The welfare impact of a fish regaining consciousness following a stun is considerable if it occurs, as the stunning method can cause physical trauma and pain in fish, and the fish may be subjected to slaughter whilst conscious (see sections 3.2.3., and 3.3.3.).

Relevant evidence:

Rainbow trout; 11/141 showed behavioural signs of consciousness following stunning (Jung-Schroers et al., 2020)^α.^

Common carp; 23.1% of the study sample showed injuries resulting from mishits (Retter et al., 2018)^µ^.

| **Likelihood of regaining consciousness before death** | | |
| --- | --- | --- |
| **Likelihood** | **Welfare impact** | **Strength of evidence** |
| Medium | High | 4+ studies (general) |
| **Relevant evidence** | | |

Likelihood:

Likelihood is medium due to the risk of mis-stuns.

Welfare impact:

The welfare impact of a fish regaining consciousness following a stun is considerable if it occurs, as the stunning method can cause physical trauma and pain in fish, and the fish may be subjected to slaughter whilst conscious (see sections 3.2.3., and 3.3.3.).

Relevant evidence:

Mixed findings;
Rainbow trout: A non-penetrative captive bolt gun abolished VERs permanently (Hjelmstedt et al., 2022)^µ^;
Sturgeon (*Acipenser spps*.): 100% juvenile sturgeon appeared irreversibly stunned (lasting at least two hrs) according to behavioural indicators (jaw relaxation and lack of opercular movement).

90% adult sturgeon appeared irreversibly stunned, and some adult individuals regained movement during exsanguination. Furthermore, only mild to moderate haemorrhage in and around the brain was detected (Gross et al., 2024)^µ^.
Channel catfish: Permanent loss of VERs in 8/10 (Hjelmstedt et al., 2025).^µ^

| **Conflicting findings between behavioural indicators and EEGs** | | |
| --- | --- | --- |
| **Likelihood** | **Welfare impact** | **Strength of evidence** |
| High | High | 2-3 studies (few spp.) |
| **Relevant evidence** | | |

Likelihood:

Whilst limited, there is evidence to suggest a high likelihood, although further research is needed.

Welfare impact:

There is a significant welfare impact if unconsciousness is inaccurately assessed (see section 3.3.4).

Relevant evidence:

Whilst limited evidence exists, behavioural, physiological and neurophysiological indicators are often contradictory:

In sturgeon, behavioural indicators (jaw relaxation, loss of opercular movement, and somatic evoked response to gill raker touch) were present in sturgeon. However, they corresponded with mild to moderate brain haemorrhage, suggesting limited insensibility (Gross et al., 2024)^µ^.

Some channel catfish restarted ventilation without recovering VERs, and some recovered VERs before they restarted ventilating (Hjelmstedt et al., 2025).^µ^
