# Supplementary material for: Stunning methods in aquaculture slaughter and their implications for fish welfare
Source: PeerJ. 2026 May 18;14:e21258. doi: 10.7717/peerj.21258 (PMC13192462; doi:10.7717/peerj.21258)
Supplement: Supplemental Information 6 — Reports likelihood, welfare impact and strength of relevant evidence across pre-stunning, induction, and loss of consciousness phases, along with details of the relevant evidence to support the synthesis presented in Table 2. [file peerj-14-21258-s006.docx]

S6: Detailed welfare assessment for Gas Mixture Narcosis in aquaculture. Reports likelihood, welfare impact and strength of relevant evidence across pre-stunning, induction, and loss of consciousness phases, along with details of the relevant evidence to support the synthesis presented in Table 2.
Key: ∞ Indicates a reference to grey literature; ^µ^ indicates a study or part of a study performed at laboratory/ research scale; and ^α^ indicates a study or part of a study performed at commercial scale.

| **Gas Mixture Narcosis** | | |
| --- | --- | --- |
| **Pre-Slaughter Phase:** | | |
| **Crowding** | | |
| **Likelihood** | **Welfare impact** | **Strength of evidence** |
| High | High | 4+ studies (general) |
| **Relevant evidence** | | |

Likelihood:

Crowding is a typical pre-stunning process, with varying degrees and duration (Daskalova, 2019; Espmark et al., 2025; Jung-Schroers et al., 2020; Rucinque et al., 2021).

Welfare impact:

Not studied explicitly in relation to this method, but the welfare impacts are well documented for multiple species of farmed fish (see section 3.1.1).

Relevant evidence:

Degree and duration of crowding can vary from farm to farm (Jung-Schroers et al., 2020).
Because of the risk of physical injury and mortality, the focus has tended to be on the impact on flesh quality, rather than the welfare of fish themselves (Lines and Spence, 2012; Stien et al., 2024). Sub-surface risks include the accidental creation of pockets when nets are tightened, trapping the fish and intensifying crowding issues (Stien et al., 2024). Technology is increasingly being used to address these issues, including the use of winch cameras or remotely operated underwater vehicles to detect sub-surface issues (Stien et al., 2024), acoustic telemetry to monitor fish behaviour (Føre et al., 2018), and stunning devices, such as in-water pipeline electrical systems that can reduce or eliminate the need for pre-stunning crowding (Welfarm, 2023). However, further research is often needed to ascertain the degree to which these methods effectively reduce stress, and other welfare impacts in fish.

| **Handling** | | |
| --- | --- | --- |
| **Likelihood** | **Welfare impact** | **Strength of evidence** |
| Variable | High | 4+ studies (general) |
| **Relevant evidence** | | |

Likelihood:

In most systems, fish are typically handled in order to be de-watered before being placed into the new controlled water. However, whilst this occurs regularly in practice, in theory it should not be required.

Welfare impact:

Not studied explicitly in relation to this method, but the welfare impacts are well documented for multiple species of farmed fish (see section 3.1.2).
Relevant evidence:

Whilst the negative impacts of handling are relatively well documented, especially in regard to flesh quality (Matos et al., 2010), they are often overlooked in the assessment of stunning systems themselves, or are grouped with crowding and other pre-slaughter stressors (Brijs et al., 2018; Matos et al., 2010). Whilst a holistic approach ensures all aspects of the stunning process are considered, there is also a need to break down the welfare implications of individual handling methods, to not only ensure that farmers can make evidence-based decisions on the most welfare-friendly options, but also to ensure targeted improvements. For instance, the development and use of rubberised nets can effectively reduce scale loss in fish (Powell, 2021).
Whilst the long-term impacts of scale loss are not relevant for fish bound for slaughter, the short-term impacts, including pain and discomfort, are highly relevant to individual fish and may also negatively impact their ability to cope with additional stressors (Lange et al., 2018; Oliveira and Galhardo, 2009; Petitjean et al., 2019). ‘Fish-friendly’ pumping systems are also under development to reduce shear force and crowding, minimise pressure fluctuations, and avoid impacts and abrasions (Krakers et al., 2015; Pan et al., 2022). However, these systems vary widely in design and effectiveness and lack validation across species. Further research and regulation are therefore needed to ensure meaningful welfare gains from the development of these technologies.

| **Air exposure** | | |
| --- | --- | --- |
| **Likelihood** | **Welfare impact** | **Strength of evidence** |
| Variable | High | 4+ studies (general) |
| **Relevant evidence** | | |

Likelihood:

In many systems, fish are first de-watered and exposed to air before going into the new controlled water. However, whilst this occurs regularly in practice, in theory it should not be required.

Welfare impact:

Welfare impacts of air exposure are well documented for multiple species of farmed fish (see section 3.1.3). Welfare impact is worsened with increasing duration of air exposure, but even a brief exposure is considered a significant welfare impact (Schuck-Paim et al., 2025).

Relevant evidence:

Whilst there is little regulatory protection for fish, best practice guidelines and certification schemes generally advise against killing fish by asphyxiation, and whilst some guidelines just state that time out of water should be minimised (European Commission, 2020; WOAH, 2015), others are more detailed. For instance, 15 seconds is increasingly being used as a limit for farmed fish, based on the behavioural responses of some fish becoming more pronounced after 15 seconds (HSA, 2016; RSPCA, 2024, 2020). However, given that there can be considerable variation between species and individuals in terms of responses to stressors, coping abilities, and coping styles, focusing solely on behavioural signs may be too limited (Castanheira et al., 2017; Erikson et al., 2016; Martins et al., 2012). Therefore, given the evidence that fish suffer severe negative affects when exposed to air for a brief time (Schuck-Paim et al., 2025), further research is urgently needed to determine humane thresholds and alternatives to current practices, including the use of stunning methods where fish are not removed from the water.

| **Induction** | | |
| --- | --- | --- |
| **Behavioural aversion** | | |
| **Likelihood** | **Welfare impact** | **Strength of evidence** |
| High | High | 1 study |
| **Relevant evidence** | | |

Likelihood:

Although based on limited findings, the method is considered to be aversive to fish. However, further research is needed.

Welfare impact:

The display of highly aversive behaviour is indicative of distress in the fish (see section 3.2.1).

Relevant evidence:

Although limited findings, it is generally considered highly aversive.

Seabream show strong acceleration of swimming and raise heads out of the water in response to CO_2_+N_2_+O_2_ and CO_2_+N_2_ gas mixtures (Roque et al., 2021)^µ^.

| **Physiological stress response** | | |
| --- | --- | --- |
| **Likelihood** | **Welfare impact** | **Strength of evidence** |
| High | Unclear | 4+ studies (general)  0 studies (method) |
| **Relevant evidence** | | |

Likelihood:

The likelihood is high based on the limited findings available, as all methods result in some degree of physiological stress response.

Welfare impact:

It is difficult to separate the stress response from pre-slaughter stressors from the narcosis.

Relevant evidence:

No relevant evidence.

| **Physical trauma** | | |
| --- | --- | --- |
| **Likelihood** | **Welfare impact** | **Strength of evidence** |
| High | Low-High | 4+ studies (general) |
| **Relevant evidence** | | |

Likelihood:

Physical trauma is considered highly likely as a result of the extreme aversive behaviours performed, which may be escalated if in crowded conditions.

Welfare impact:

The welfare impact varies depending on the scale of the trauma and the duration the fish is conscious for (see section 3.2.3).

Relevant evidence:

No specific evidence was found in the literature in relation to the method, but aversive struggling behaviours can result in physical injuries.

| **Loss of Consciousness and Recovery Risk** | | |
| --- | --- | --- |
| **Risk of delayed onset of unconsciousness** | | |
| **Likelihood** | **Welfare impact** | **Strength of evidence** |
| High | High | 1 study |
| **Relevant evidence** | | |

Likelihood:

Although limited research, unconsciousness is not immediate.

Welfare impact:

The welfare impact of delayed unconsciousness is significant as the fish show extreme aversion before unconsciousness (see sections 3.2.1 and 3.3.1).

Relevant evidence:

Seabream exposed to a gas mixture of CO_2_+N_2_+O_2_ lost posture and balance at 01:12 ±0:32s, and fish exposed to CO_2_+N_2_ at 01:23±0:32s (Roque et al., 2021)^µ^.

| **Risk of failed induction of unconsciousness** | | |
| --- | --- | --- |
| **Likelihood** | **Welfare impact** | **Strength of evidence** |
| High | High | 1 study |
| **Relevant evidence** | | |

Likelihood:

Unconsciousness is not immediate with gas mixture narcosis, and so it is considered a ‘failed induction’.

Welfare impact:

The welfare impact of failed unconsciousness is significant as the fish show extreme aversion before unconsciousness (see sections 3.2.1 and 3.3.2).

Relevant evidence:

Limited evidence suggests narcosis is not immediate (Roque et al., 2021)^µ^.

| **Likelihood of regaining consciousness before death** | | |
| --- | --- | --- |
| **Likelihood** | **Welfare impact** | **Strength of evidence** |
| Unknown | High | 0 studies |
| **Relevant evidence** | | |

Likelihood:

This area is understudied, and the likelihood depends on how long the fish are kept in the water (see section 5.3.2).

Welfare impact:

The welfare impact of a fish regaining consciousness following narcosis can be considerable, as they are then conscious during the following slaughter processes (see section 3.3.3).

Relevant evidence:

Dependent on the duration of narcosis, the method is not immediate, and so some individuals may be killed during the process, whereas others may not have reached unconsciousness.

| **Conflicting findings between behavioural indicators and EEGs** | | |
| --- | --- | --- |
| **Likelihood** | **Welfare impact** | **Strength of evidence** |
| Unknown | High | 4+ studies (general)  0 studies (method) |
| **Relevant evidence** | | |

Likelihood:

Limited research in this area has been conducted to determine the likelihood.

Welfare impact:

There is a significant welfare impact if unconsciousness is inaccurately assessed (see section 3.3.4).

Relevant evidence:

No research was found in this context, although conflicting findings are found for other methods, including the use of CO_2,_ which may be generalisable.
